# Exosome-based strategies for diagnosis and therapy of glioma cancer

**DOI:** 10.1186/s12935-022-02642-7

**Published:** 2022-08-21

**Authors:** Mohsen Karami Fath, Jalil Azami, Alireza Masoudi, Reza Mosaddeghi Heris, Elnaz Rahmani, Fatemeh Alavi, Armina Alagheband Bahrami, Zahra Payandeh, Bahman Khalesi, Masoomeh Dadkhah, Navid Pourzardosht, Vahideh Tarhriz

**Affiliations:** 1 Department of Cellular and Molecular Biology, Faculty of Biological Sciences, Kharazmi University, Tehran, Iran; 2 Faculty of Veterinary Medicine, Urmia University, Urmia, Iran; 3 Department of Laboratory Sciences, Faculty of Alied Medical Sciences, Qom University of Medical Sciences, Qom, Iran; 4 Neurosciences Research Center, Tabriz University of Medical Sciences, Tabriz, Iran; 5 Department of Clinical Pharmacy, Faculty of Pharmacy, Kerman University of Medical Sciences, Kerman, Iran; 6 Department of Pathobiology, Faculty of Specialized Veterinary Sciences, Science and Research Branch, Islamic Azad University, Tehran, Iran; 7 Department of Biotechnology, School of Advanced Technologies in Medicine, Shahid Beheshti University of Medical Sciences, Tehran, Iran; 8 Department Medical Biochemistry and Biophysics, Division Medical Inflammation Research, Karolinska Institute, Stockholm, Sweden; 9 Department of Research and Production of Poultry Viral Vaccine, Razi Vaccine and Serum Research, Tabriz, Iran; 10 Pharmaceutical Sciences Research Center, Ardabil University of Medical Sciences, Ardabil, Iran; 11 Biochemistry Department, Guilan University of Medical Sciences, Rasht, Iran; 12 Molecular Medicine Research Center, Biomedicine Institute, Tabriz University of Medical Sciences, Tabriz, Iran

**Keywords:** Glioblastoma multiforme, Exosome, Immunotherapy, MicroRNA, Biomarkers

## Abstract

Glioblastoma belongs to the most aggressive type of cancer with a low survival rate that is characterized by the ability in forming a highly immunosuppressive tumor microenvironment. Intercellular communication are created via exosomes in the tumor microenvironment through the transport of various biomolecules. They are primarily involved in tumor growth, differentiation, metastasis, and chemotherapy or radiation resistance. Recently several studies have highlighted the critical role of tumor-derived exosomes against immune cells. According to the structural and functional properties, exosomes could be essential instruments to gain a better molecular mechanism for tumor understanding. Additionally, they are qualified as diagnostic/prognostic markers and therapeutic tools for specific targeting of invasive tumor cells such as glioblastomas. Due to the strong dependency of exosome features on the original cells and their developmental status, it is essential to review their critical modulating molecules, clinical relevance to glioma, and associated signaling pathways. This review is a non-clinical study, as the possible role of exosomes and exosomal microRNAs in glioma cancer are reported. In addition, their content to overcome cancer resistance and their potential as diagnostic biomarkers are analyzed.

## Introduction

Glioma is one of the most prevalent primary tumors of the central nervous system (CNS) which is identified by the signs such as high aggression, relapse, and mortality. Approximately half of all gliomas are glioblastoma multiform (GBM) [1, 2]. Moreover, stem cell therapy has been proposed as the other novel therapeutic option for glioma. In adults, glioma is categorized as the most common and highly aggressive brain tumor. In preclinical works, several factors such as hormones, cytokines, proteases, and chemokines are secreted by stromal cells to modulate the tumor microenvironment [3].

The lack of a non-invasive method is the major problem to assess the GBM treatment. This issue hinders the effective clinical management of the GBM. The gliomas prognosis could be very poor owing to numerous reasons. First, few drugs exist with good therapeutic effects in clinical practice. Most of the drugs are rendered ineffective by the permeation restriction exerted by blood–brain barrier (BBB) and the higher drug resistance of glioma. On the other hand, different limitations such as poor stability, toxic side effects, and immune activation exacerbate the situation. Deal with this tumor becomes even more difficult considering its high epigenetic and genetic heterogeneity [4, 5]. Glioma is a heterogeneous complex tumor, which comprises tumor cells and numerous non-tumor cell types, including astrocytes, endothelial cells microglia, and immune cells that establish the complex glioma microenvironment.

Exosomes are a new class of nano-sized extracellular vehicles (EVs) released by the vast majority of cell types both in vivo and ex vivo. They are discovered by advances in high-resolution imaging within the cell secretome. Exosomes are secreted from cells and released into surrounding body fluids upon fusion of multivesicular bodies and the plasma membrane. However, they were initially proposed as cellular waste resulting from cell damage, or by-products of cell homeostasis, they are endowed with the capability of cell reprogramming and data trafficking. Moreover, they are shown to contribute to the transduction of pathophysiological conditions [6, 7].

The present study is focused on the general structure of exosomes and their involvement in the initiation or progression of glioma cancer. Since key features of exosomes are strongly dependent on the origin cells and development status, it is essential to review key modulating molecules, clinical relevance to glioma, and associated signaling pathways. Moreover, evidence on exosome contents is shared, as a potent diagnostic biomarker, which exerts a possible role in chemo-resistance after exosome-based therapies. Finally, we reviewed the current knowledge on glioma cancer immunotherapy particularly dendritic cells (DCs), mRNA vaccines, and bi-specific antibodies.

## Exosomes and extracellular vesicles

As EV properties improve, markers such as protein and lipid continue to be very useful to demonstrate the generic structure of EVs. Based on EVs derived from mammalian vs non-mammalian vs non-eukaryotic cells, or claims, which are generic to all types of EVs, or instead specific to subtypes of EVs, the markers applied for the characterization EVs may differ. The EV-TRACK knowledgebase is approved by ISEV to represent and enhance accuracy and reproducibility in EV related studies, according to the MISEV guidelines. With regards to exceptions to each rule, MISEV2018 is meant to guide and developed the field, not stifle it [8]. Additionally, glioma cells (GMs) enhance glucose consumption and lactate production via increasing the levels of monocarboxylate transporter 1 (MCT1) and the cluster of differentiation 147 (CD147) and their localization at the plasma membrane to remove intracellular lactate out of cells to maintain continuous glycolysis. This results in the accumulation of lactate in the tumor microenvironment (TME) [9]. Exosomes derived from GMs with a size ranging approximately from 30 to 200 nm can spread into systemic bio-fluids, such as cerebrospinal fluid (CSF) and blood by crossing the blood-CSF barrier (BCSFB) and the blood–brain barrier (BBB). However, GMs-derived exosomes have been identified as great platforms for the discovery of effective biomarkers for glioma progression. In addition to magnetic resonance imaging (MRI) and computed tomography (CT) scans, intracranial biopsies have been proposed for the diagnosis and prognosis of glioma [10]. Due to excellent improvement in sensitivity, the TiO2-CTFE-AuNIs real-time label‐free plasmonic biosensor demonstrated a good potential in detecting of prognostic biomarkers in GMs-derived exosomes for application in glioma liquid biopsy [11]. Exosomes derived from glioma which releases a great number of them in the tumor microenvironment (TME), are a type of extracellular vehicles (EVs) with a size range of 30–200 nm, and aggregates in a wide range of extracellular milieu and bio-fluids including blood, urine, and cerebrospinal fluid, with a crucial role in cell–cell communication [12]. In particular, GMs-derived exosomes can cross the BBB and are found in peripheral circulation [13] which makes them as an important useful biomarker discovery platform for monitoring glioma progression. BIGH3 is one of the promising ones as the prognostic biomarkers of glioma and especially has been identified in GMSs derived exosomes [14].

Mesenchymal stem cells (MSCs) are one of the elements of the tumor microenvironment (TME) which plays a key role in primary tumor growth and metastasis. For example, exosomes secreted by MSC-differentiated adipocytes have been proposed that can promote EMT in breast cancer cells via activation of the Hippo signaling pathway [15]. Similarly, studies in lung cancer confirm that exosomes secreted by hypoxic bone-marrow-derived mesenchymal stem cells (BMSCs) in the tumor microenvironment promote cancer cell invasion and EMT through transfer of miR-193a-3p, miR-210–3p, and miR-5100 from hypoxic BMSCs to cancer cells [16]. Similarly, exosomes secreted by hypoxic bone-marrow-derived mesenchymal stem cells (BMSCs) in the tumor microenvironment can promote lung cancer cell invasion and EMT by miR-193a-3p, miR-210–3p, and miR-5100 transferring from hypoxic BMSCs to lung cancer cells [16].

A large number of strategies have been developed for isolating EVs including density gradient centrifugation, immunomagnetic bead-based extraction, chromatography, ultrafiltration, and microfluidic device [17]. For increasing the efficacy of anticancer agent delivery to glioma and also to maintain the effective drug-level in glioma tissues, it’s crucial to enhance the loading efficiency of an anticancer drug into exosomes. For this reason, improving the loading efficiency of therapeutic agents into exosomes needs to develop various methods, including electroporation, incubation, and chemical reagents, have been investigated [18]. By developing the micro- and nanofabrication technologies, microfluidics application to drug loading and delivery to cells has been investigated [19]. However, the loading of a drug in exosomes via the microfluidics-based approach demonstrated that microfluidics could represent a better performance in loading a drug in cells better than the other methods. This technique due to its capability of controlled and precise setup could be easily ordered to achieve enhanced drug loading [20]. Microfluidics represented several advantages, including simple and efficient setup, parameters that can be controlled such as flow rate and pressure, to maintain optimal conditions for drug loading. Loading of drug-assisted by microfluidics into liposomes in the treatment of various cancers has been investigated for several decades elsewhere [21].

## Classification of glioma cancer

Based on the world health organization (WHO) criteria, glioma is histopathologically classified into 4 grades; The main glioma types include pilocytic astrocytoma (grade I), anaplastic astrocytomas (grade II), oligodendrogliomas (grade III), and glioblastomas or isocitrate dehydrogenase (IDH) (grade IV) as the most progressive and lethal subtype of glioma with inferior prognoses [22, 23]. Several mutations have been identified within IDH that typically involves young patients who have prior second or third glioma tumors [24]. Most studies have indicated that the mutations occur at arginine 132 of IDH1 and the homologous arginine 172 of IDH2 [25].

## IDH-mutant astrocytic gliomas

Probably, IDH mutations are the initial genetic aberrations occurring in a developing glioma. Nevertheless, data from the mice model showed that IDH mutation is not adequate for tumorigenesis [26, 27]. Additional mutations are usually found in IDH-mutant astrocytomas within ATRX and TP53. ATRX mutation results in detectable loss of nuclear expression. This mutation plays a vital role in chromatin remodeling and regulation of telomere length [28]. There are different genetic alterations related to progression from diffusing to anaplastic astrocytoma, and ultimately IDH-mutant glioblastoma. Such changes involve chromosomal 9p21 deletions (including CDKN2B (encoding cyclin-based kinase inhibitor B called p15^INK4B^), CDKN2A (encoding cyclin-based kinase inhibitors 2A called p16^INK4A^, as well as ARF called p14ARF), 19q deletion, and various other chromosomal imbalances [27, 29].

## IDH-mutant and 1p/19q-co-deleted oligodendroglial tumors

Genetically, IDH mutation co-exists with whole-arm co-deletion of 19q and 1p chromosome, which refers to oligodendrogliomas. An imbalanced t(1;19) (q10;p10) translocation [30] leads to the latter co-deletion and better prognosis for oligodendrogliomas.

In more than 95% of such tumors, activating mutations are observed in the TERT-promoter area, which results in aberrant expression of telomerase reverse transcriptase. Moreover, the CIC mutation is detectable in more than 2/3 of patients, which inactivates the *Drosophila* capsicum protein homolog (a transcriptional repressor) [31]. Approximately 1/3 of oligodendroglial tumors contain FUBP1 mutations (encoding far upstream element-binding protein 1, included in the regulation of MYC expression) [32]. NOTCH1 is the gene responsible for encoding the epigenetic regulators like SETD2 and phosphatidylinositol 3-kinase (PI3K) pathway genes such as PIK3CA [33]. Genetic alterations which are connected to a phenotype of the prevalent aggressive disease include the 9p21 deletions, activation of MYC signaling, and transcription factor 12 (TCF12) related mutations [34].

## IDH-wild-type glioblastoma

In all age ranges people can be affected by IDH-wild-type glioblastomas, however, they occur predominantly in patients who have more than 50 years old. These tumors appear typically as ‘primary glioblastoma’. The characteristics of lower-grade IDH-wild type glioblastomas in adult patients are similar to IDH-wildtype glioblastoma including the homozygous deletion or mutation of phosphatase and tensin homolog (PTEN), TERT-promoter mutations, monosomy of chromosome 10, homozygous deletion of CDKN2A, and CDKN2B [35, 36]. Mutations in the regions encoding for PI3K-regulatory subunit 1 (PIK3R1), TP53, PIK3CA, and neurofibromatosis type 1 (NF1) have been proposed as less-common changes. Amplification of Platelet-Derived Growth Factor Receptor Alpha (PDGFRA), EGFR, and MET genes is also observed in IDH-wild-type glioblastomas. The CDK4 and CDK6 cyclin-based kinase genes mediate the transition of the G1 phase of the cell cycle into the S phase; the MDM2 and MDM4 gene, encode proteins that inhibit the p53 activity [35, 37]. In almost forty percent of IDH-wild-type glioblastomas, EGFR amplification is observed, and 1/2 of these tumors also harbor a genetic rearrangement which leads to the removal of EGFR exons 2–7 [35, 37]. A druggable mutant protein is encoded by BRAF-V600E, which is found in about 50% of epithelioid glioblastomas [37].

## IDH-mutant glioblastoma

This type of gliomas shares a similar molecular profile as IDH-mutant astrocytomas such as frequent ATRX and TP53 mutations, along with a G-CIMP53. Compared to G-CIMP-positive and IDH-mutant astrocytic gliomas, lower DNA methylation levels are found in a subset of patients, which is correlated to adverse outcomes [38].

## Glioblastomas and progressive gliomas

H3K27M mutant diffuse midline glioma is typically located in the thalamus, spinal cord, or brain stem [39]. The genetic hallmark of such tumors is the K27M mutation in HIST1H3B/C or the histone-H3-encoding genes H3F3A [40]. These mutations globally reduce the cellular trimethylation of histone H3 at lysine 27 (H3K27me3) through PRC2 impaired recruitment and inhibiting the histone-lysine N-methyltransferase EZH2 [41, 42]. Activin A receptor type 1 (ACVR1) gene mutations are carried via 20% of DIPGs; however, FGFR1 alterations are mainly associated with thalamic tumors [43, 44].

## Glioma molecular biomarkers

According to the WHO classification of glioma (as of 2016), C11orf95–RELA fusion, IDH1/2 mutation, H3-K27M mutation, and 1p/19q deletion are categorized as diagnostic biomarkers defining distinct glioma entities. Further diagnostic information could be provided by other biomarkers such as nuclear ATRX expression loss, BRAF fusion or mutation, TERT-promoter mutation, and H3-G34 mutation [36]. Examples of gene mutations documented in glioma are listed in Table 1. Several predictive biomarkers are described for glioma patients, which could be helpful in anti-glioma treatment planning and prognosis. In this regard, the methylation of MGMT-promoter predicts the benefits from chemotherapy with alkylating-agent in IDH wild kind glioma patients, chiefly in older patients [45, 46]. This methylation typically happens homogeneously within various sites in the patients with glioma. Nonetheless, secondary temozolomide resistance could be developed by tumors with the methylation of MGMT-promoter due to the mutations which drive tumor recurrence and clonal evolution [47]. For instance, a hypermutator genotype can be raised by mutations in DNA-mismatch-repair genes. Regardless of the clinical significance of the methylation of MGMT-promoter, there are still challenges in diagnostic testing of this genetic change. Heterogeneous methylation of MGMT-related CpG sites within various tumors, give rise to uncertain thresholds for positive results in tumors with methylation. This uncertainty promotes the borderline or poorly discoverable MGMT, and exploitation of non-standardized diverse examining approaches [48]. The 1p/19q co-deletion independently predicts the benefits of adding PCV chemotherapy to upfront irradiation treatment in patients with anaplastic glioma. A decent survival rate is reported in the subjects with 1p/19q co-deletion and those with IDH-mutant gliomas. There is little information on the basic molecular mechanisms of this favorable treatment response [49]. Hu et al. used the Meta-ANOVA method to evaluate the effect of 1p/19q co-deletion on OS and PFS by synthesizing the results in multivariable analyses in previous studies. Their results indicate that 1p/19q co-deletion had a significant protective effect on the prognosis of II and III oligodendrogliomas. Patients having 1p/19q co-deletion and without IDH-1 mutation have a 91% reduction in the hazard of death compared to patients without co-deletion and with IDH-1 mutation. After adjusting for age, the extent of resection, and adjuvant therapy, patients with 1p/19q co-deletion and a total resection have an 81% reduction in hazard of mortality compared to patients without both co-deletion and total resection. In addition, patients with 1p/19q co-deletion and younger age (less than or equal to 40) have a 71% reduction in the hazard of death compared to patients with no 1p/19q co-deletion and older than 40 years [50].

**Table 1:** Examples of gene mutations implicated in glioma

Molecular Markers	Type of mutation and alteration	Type of tumor	Sample	WHO grade	Prevalence in patients (AACR)	Refs.
1p/19q	Deleting long arm of Ch. 19 and short arm Ch. 1	Oligodendrogliomas	360 patients	II, III	12.5%	[194]
Atrx	Deletion	Low grade and secondary GBM and	Glioma patients	I, II	2.42%	[195]
BRAF	Fusion gene KIAA1549:BRAF	Pilocytic Astrocytomas	–	III	8.47%	[196]
CDK4	Amplification	Proneural	–	IV	3.19%	[197]
IDH	Missense mutation at arginine 132 or 172	Secondary glioblastoma and oligodendrogliomas	149 GBMs	II, III, IV	25.88%	[194]
MET	Amplification	Mesenchymal	Glioma patients	III, IV	3.12%	[198]
MGMT	Promoter methylation	Glioblastoma and Low–Grade Gliomas	52 patients	I, II	3.21%	[197]
NF1	Deletion	Mesenchymal and Pilocytic Astrocytoma	–	I	14.96%	[197]
PDGFR	Amplification	Proneural	GBM patients	–	7.66%	[199]
PTEN	Deletion	Glioblastoma	–	IV	21.97%	[197]
PI3K	Activation mutation	Glioblastoma	–	IV	14.7%	[197]
TERT	Promoter methylation	Primary GBM and Oligodendroglioma	3,477 patients	II, III	32%	[200]
H3F3A	H3‑K27 trimethylation	Pediatric (children)	Glioma patients	IV	3.69%	[195]

## Role of exosomes in the detection and treatment of glioma cancer

**Fig. 1:**
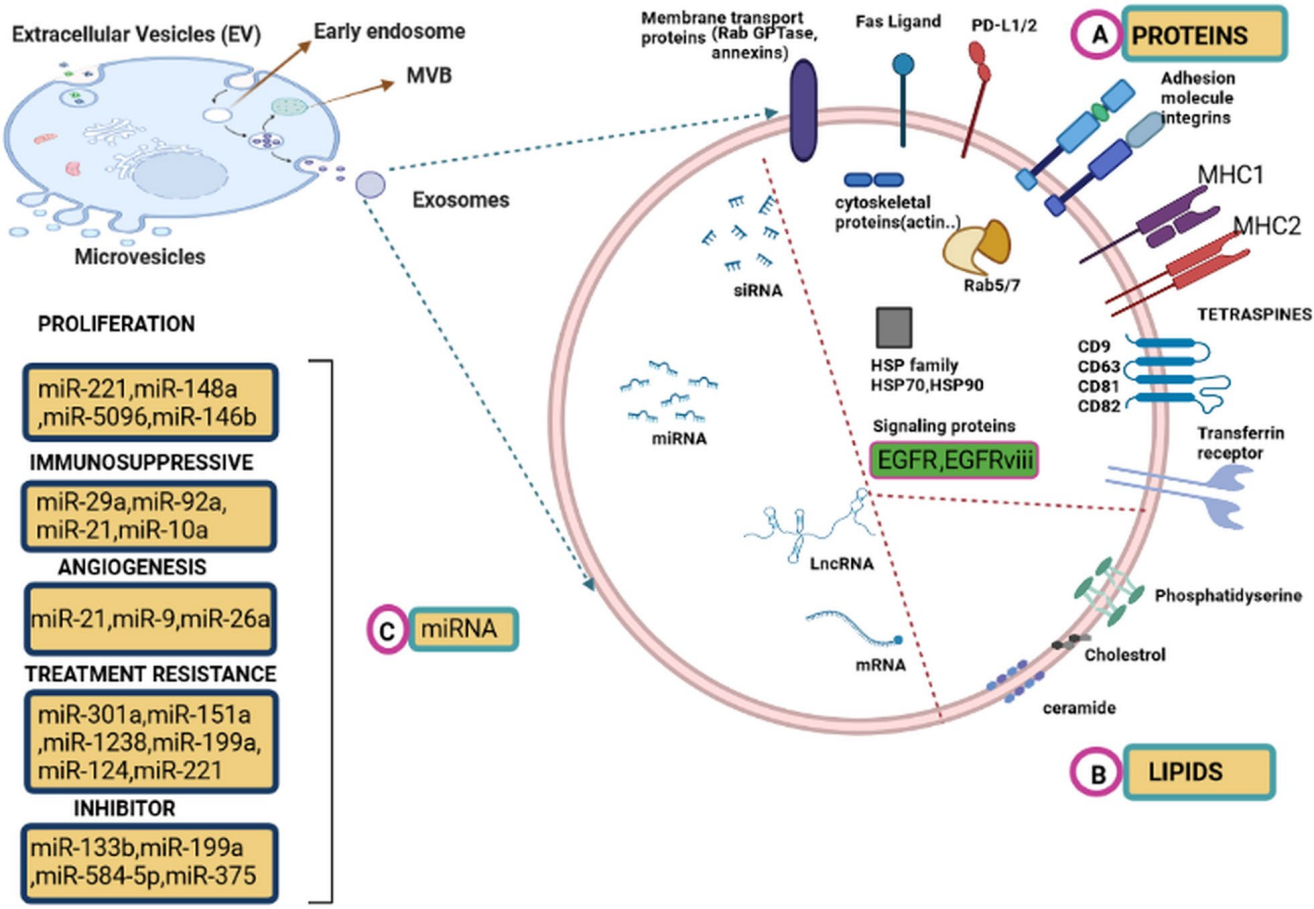
The exosome and exosomal microRNA role in glioma therapy

Exosomes play a key role in the establishment and evolution of intercellular signaling pathways [51–54]. The primary proteins on the endosomal vesicles including the CD9, CD81, and CD82, could also be found on the surface of the exosomes. These extracellular vesicles could carry nucleic acids, proteins, lipids, and metabolites. Intraluminal vesicles (ILVs) are eventually released as exosomes (~ 40 to 160 nm in diameter) via exocytosis following MVB fusion to the plasma membrane [55]. This process results in the net synthesis of a mixed population of exosomes for each cell throughout time. Exosome-derived miRNAs are the most abundant and essential biomolecules that play a key role in tumor control [56–58]. The presence of a large number of circulating exosomes, as well as exosomal cargo in the cancer microenvironment indicate that these subcellular secretory nanoparticles may play a role in the creation of intricate cross-talk systems in tumor initiation, development, and dissemination (See Fig. 1 for more details).

Due to the wide range of heterogeneity and complexity of morphology, molecular treatment and classification of GBM is an intricate process [59]. Recently, research regarding the role of exosomes in the formation of gliomas has garnered a lot of attention [60]. Tumor angiogenesis and migration [61] are promoted by the production of glioma exosomes, proliferation of Glioma cells, [62], regulation of tumor intrusiveness, and induction of signaling pathways. Incidence, progression, and treatment unresponsiveness of glioma are all affected by GDEs. Tumor cells, fibroblasts, and immune cells, which compose the tumor microenvironment (TME), are all involved in the resistance of glioma to different therapies [63]. Inside the TME [64], cellular communication between the tumor and its surrounding is primarily done by mixtures of microbubbles made of cellular membrane or originating cells. Exosomes play significant functions in the immunosuppression, induction, intrusion, metastasis, and treatment unresponsiveness of cancers [52, 65]. Exosomes derived from U251 human glioma cells include diverse proangiogenic elements which enhance endothelial cells (ECs) growth, immigration, and lumen construction [65]. Furthermore, GDEs control the angiogenic ability of EC by increased or decreased expression of miRNA. Xu et al. have declared that the angiogenic potential of ECs is induced by exosomes produced by glioma stem cells (GSC). These exosomes boost the levels of miR-21, proangiogenic growth factor, and vascular endothelial growth factor (VEGF) [66]. Moreover, Yue et al. have reported that the sensitive cells lose their radiation sensitivity due to the secreted exosomal miR-301a from GBM cells in hypoxic conditions. This property is rooted in the ability of miR-301a to regulate the Wnt/β-catenin pathway and targeting of anti-oncogene TCEAL7. Therefore, the Exo-miR-301a/TCEAL7 signaling pathway could become a novel target to overcome the radiotherapy resistance in GBM patients [67]. Based on the study conducted by Zeng et al., exosomal miRNAs may contribute to TMZ resistance in GBM cells [68]. The expression of miR-151a was measured in two GBM cell lines, which are resistant to TMZ. Increased resistance to TMZ is correlated to the lower expression of miR-151a. The extent of chemo-resistance within the GBM tumors is correlated with the number of exosomes that contain the miRNA-151a in CSF. Thus, one non-invasive strategy to assess the extent of chemo-resistance would be the sampling of exosomal miR-15 as ‘liquid biopsy’. Exosomes could be introduced as a new approach to treat the refractory GBM tumor [68]. MiR-155HG/miR-155 is a vital factor in the progression of GBM, and suppressing this factor by NSC141562 might be used for GBM treatment [69]. Shi et al. have found that one approach to impede the growth of non-small cell lung cancer could be the targeting of LHX2 by miR-1238, which acts as a tumor inhibitor [70]. Yin et al. have revealed that over expressed miR-1238 has a key role in the resistance acquired against TMZ in GBM patients [71, 72]. Aside from the prognostic significance of miR-1238 as a biomarker of tumor chemotherapy evaluations, it could be a new target to treat GBM [71, 72]. Recent research has demonstrated that the miR-5096 could make glioma cells become more invasive. MiR-5096 could trigger the generation of filamentous pseudopodia via modulation of the K + channel Kir4.1. It also could increase the exosome secretion, which could result in GBM metastases [73]. GBM progression risk might be enhanced by miR-148a according to The Cancer Genome Atlas (TCGA) [74]. The rate of GBM development might be elevated by mir-148a through increased signaling of CADM1/STAT3 [75, 76]. Cai et al. have discovered that the body fluids of GBM patients had a higher number of exosomes carrying the miR-148a [77]. Progression and metastasis of T98G cancer cell line were inhibited by suppressing miR-148a. Moreover, the results of a luciferase test showed that miR-148a could target CADM1. In samples obtained from GBM patients, a close correlation was observed between the upregulation of CADM1 and the exosomal miR-148a. MiR-148a antagonist elevates the STAT3 protein levels, which promote the activation of STAT3 signaling. Ultimately, they discovered that exosomes carrying the miR-148a could, activate STAT3 signaling through CADM1 and could promote tumor progression and metastasis. They suggested that GBM prognosis and treatment could be performed using and targeting the miR-148a [77]. Guo et al. have found that glioma cell in hypoxic condition releases exosomes, which contain miR-29a. It has been shown that miRNA containing exosomes (generated by gliomas) could make an immunosuppressive microenvironment in the tumor. Moreover, they also explained the regulatory mechanism behind the functional induction of MDSCs by exosomal miR-29a/miR-92a-based modulated [78]. MiR-21 is an interesting candidate to be targeted for GBM treatment and a well-known tumorigenesis inducer. Increased apoptosis rate and radio-/chemo sensitivity of tumor and decreased tumor proliferation are observed following the miR-21 suppression [79–83]. The possible effects of downregulated miR-21 on C6 glioma tumor cell lines are investigated by Monfared et al., [84].

GBM can also be inhibited by miR-133b [85]. The expression of exosomal miR-301a in serum of glioma patients has been evaluated compared to healthy cases. It has been observed that exosomal miR-301 is overexpressed in the patients. the amount of exosomal miR-301a in serum could be a proposed indicator of variations in glioma patients [86]. Manterola et al. have shown that the presence of exosomal miR-301a in sera could be a useful biomarker in GMB diagnosis and prognosis. They have evaluated the expression of exosomes extracted from the serum of 60 people, which were divided into healthy control and GMB patients by miRNA chip technology. They have shown that miR-564-3p, miR-320, and RUN6–1 had the highest variation, and RUN6–1 alone or both miRNAs and RUN6–1 could be utilized for the detection of GBM [87]. Their research also confirmed that miRNAs containing exosomes that are derived from cancer cells and sera could be used as a biomarker for prognoses and evaluation of CNS cancer [88]. Role of different exosomal microRNA in Glioma summarized in Table 2.

**Table 2:** Role of different exosomal microRNA in glioma

Exosomal microRNA	Sample	Target	Note (result)	Expression status	Refs.
miR-301a	Serum of glioma patients	TCEAL7	Migration of β-catenin from cytoplasm to the nucleus was impeded by TCEAL7 which resulted in regulation of Wnt/β-catenin pathway and so TCEAL7 could be tumor suppressor in GMB	up	[67]
miR-151a	GBM patients	XRCC4	Decreasing the amount of XRCC4, postponing the clearance of DSB and increase the sensitivity of cells to TMZ was induced by miR-151a	up	[68]
miR-1238	Human tissue samples (GBM specimens)	CAV1	Absence of miR-1238 can increase sensitivity of resistant GBM cells through CAV1/EGFR pathway	up	[71]
mir-5096	Human microvascular endothelial cells (HMEC) & Glioblastoma cells U87-U251	Kir4.1, AQP-4	In gliomas, miR-5096 was found to be downregulated	down	[73]
miR-148a	Human glioblastoma cell line T98G	CADM1	STAT3 signaling pathway was activated by miR-148a through targeting CADM1 and stimulates proliferation and metastasis	down	[77]
miR-29a	10-week-old male C57BL/6 mice/ Myeloid-derived suppressor cells (MDSCs)	Hbp1	In gliomas, miR-29a expression was increased	up	[78]
miR-92a	10-week-old male C57BL/6 mice/ Myeloid-derived suppressor cells (MDSCs)	Prkar1a	Up regulations of miR-92a was seen in gliomas	up	[78]
miR-133b	Human brain tissues (Normal & glioma)	EZH2	EZH2 and the Wnt/β-catenin signaling pathway inhibiting by miR-133b which lead to suppression of proliferation, invasion, and migration	down	[201]
miR-199a	Human brain tissues (Normal & glioma)	AGAP2	miR-199a decreased expression of AGAP2which lead to suppressing of glioma development	down	[202]
miRNA-584-5p	U87 human glioma cells	CYP2J2	miRNA-584-5p lessen the growth and intrusion of glioma cells	up	[203]
miR-9	Glioma patient specimens & glioma cell lines	COL18A1, THBS2, PTCH1 and PHD3	angiogenesis was elevated by miR-9	up	[204]
miR-10a and miR-21	glioma patients	RORA, PTEN	expression of miR-10a and miR-21 stimulated by hypoxia in GDEs facilitates MDSC growth and activation via targeting RAR-related orphan receptor alpha (RORA) and phosphatase and tensin homolog (PETN)	up	[205]
miR-21, miR-222 and miR-124-3p	Blood samples of glioma patients	–	Expression of miR-21, miR-222 and miR-124-3p were decreased in gliomas	up	[206]
miR-125b	Tumor samples of GBM patients	–	Regulation of miR-125b increased in gliomas	up	[207]
mir-21	Human brain endothelial cells	VEGF	in gliomas expression of mir-21 increased	up	[66]
miR-124a	Bone marrow–derived mesenchymal stem cells	FOXA2	unusual lipid build-up inside cells due to silencing of FOXA2 by miR-124a	up	[208]
miR-451, miR-21	primary human glioblastoma	c-Myc	-	up	[209, 210]
miR-221	Human umbilical vein endothelial cells (HUVECs)	–	miR-221 expression in gliomas elevated	up	[211]
miR-21, miR-103, miR-24, and miR-125	serum and cerebrospinal fluid of glioblastoma patients	–	Expression of miR-21, miR-103, miR-24, and miR-125 in gliomas were increased	up	[212]
miR-302–367	GSCs primary cell lines TG1, TG6 and GB1	CXCR4/SDF1, SHH, cyclin D, cyclin A and E2F1	Cells which are adjusted to GBM internalized exosomes with high levels of miR-302–367	up	[213]
miR-1290, miR-1246	patient-derived primary cells	–	Expression of miR-1290 and miR-1246 in gliomas increased	up	[214]
miR-1587	glioma-associated human mesenchymal stem cells (GA-hMSC) & glioma stem-like cells	NCOR1	Expression of nuclear receptor co-repressor NCOR1which is tumor suppressor reduced by miR-1587	down	[215]
miR-375	human marrow stromal cells (hMSCs)	SLC31A1	While apoptosis promoted, immigration and intrusion and proliferation are inhibited by miR-375and suppression of SLC31A1development of glioma cell impeded	down	[216]
miR-1246	CSF of GBM patients	TERF2IP	The STAT3 signaling pathway is activated by miR-1246, while the NF-B signaling pathway is inhibited	down	[217]
miR-124	GBM patients	CDK6	GBM cell movement is reduced by miR-124	down	[218]
miR-328-3p, miR-339-5p, miR-340-5p, miR-485and miR-543 -3p	serum specimens of patients with glioma tumors	–	Expression of miR-328-3p, miR-339-5p, miR-340-5p, miR-485-3p, and miR-543 were increased in glioma	up	[13]
miR-182-5p, miR-486-5p	serum specimens of patients with glioma tumors	–		down	[13]
miR-454-3p	serum and tissue samples of glioma patients	ATG12	Immigration, intrusion and autophagy are inhibited in glioma by miR-454-3p	down	[219]
miR-146b	rat model of primary brain tumor	EGFR and NF-κB	EGFR and NF-κB protein are reduced by miR-146b in 9 L glioma cells in vitro	down	[220]
miR-301a	GBM patients	PTEN	expression of PTEN increased by miR-301a	up	[86]
miR-221	glioma patients	DNM3	expression of DNM3 increased by miR-221	up	[221]
miR-26a	tissue samples of glioma patients	PTEN	Proliferation and angiogenesis are boosted by miR-26a	up	[222]

## Exosome for regulation of Immune response in cancer and glioma

Most cancer cells express CD47 on the surface; CD47 is known to bind to signal regulatory protein α (SIRPα). Binding CD47 with SIRPα on innate immune cells, such as macrophages and dendritic cells, initiate the “don’t eat me” signal that blocks phagocytosis and causes tumors to escape from phagocytosis [89]. Exosomes have also been engineered for increasing phagocytosis of cancer cells by macrophages. “don’t eat me” signal was achieved using CD47-expressing exosomes originating from human serum to further decrease nanocarrier clearance by the MPS. The immunocyte-derived exosomes express CD47 receptor on their surface that interacts with SIRPα which triggers the “Don’t-Eat-Me” signal & blocks their consumption by phagocytes. Koh et al. [90] found that exosomes from SIRPα variant-transfected HEK293T cells enhanced the phagocytosis of CT26.CL25 and HT29 tumor cells by macrophages and enhanced T-cell infiltration via antagonizing the interaction between CD47 and SIRPα, thereby inhibiting the growth of cancer in syngeneic mice models.

## Correlations between PTEN-containing exosomes and GBM

PTEN has a critical role in the signaling pathway of PI3K [91]. An interesting issue about PTEN is its subcellular localization and its migration [92]. In various circumstances, such as cell differentiation and cell cycle arrest, PTEN localizes in the nucleus in addition to the cytoplasm [93, 94]. Stress and apoptosis-induced factors promote the PTEN build-up in the nucleus [95, 96]. Unlike their role in the plasma membrane and phosphatase dependent functions, enhanced chromosomes stability is associated with nucleus dependent functions of PTEN [97]. Tumor invasiveness is correlated to the absence of PTEN in nucleus, which implies the inhibitory processes of PTEN accumulation in the nucleus [97–100]. The PI3K/AKT/mTOR is one of the central molecular pathways in the development of GBM. This pathway is generally suppressed by PTEN. Putz et al. have indicated that as a result of excessive tumor growth [101], the contents of exosomes are not limited to miRs [102]. PTEN trafficking is suggested to be supported by exosomes. Generally, PTEN accumulates in nucleus or cell cytoplasm. A correlation between lack of nucleus PTEN and aggressiveness of tumor has been suggested. Migration of PTEN via exosomes is necessary to maintain the tumors-free status. Internalization of exosomes, which contain higher amounts of PTEN, is promoted by Ndfip1 protein. Since Ndfip1 is suppressed in GBM, PTEN is inhibited from accumulation in the nucleus. This condition leads to boosted tumor cell survival and growth [103]. Other fundamental elements which facilitate the proliferation of GBM include human epidermal growth factor receptor 2 (HER2), EGFRvIII, and PDGFR. Transfer of exosomes enriched with HER2, EGFRvIII, and PDGFR to unaffected cells stimulates tumorigenesis [103]. The functions of PTEN-containing exosomes in GBM cell development are summarized in Table 3.

**Table 3:** The functions of PTEN-containing exosomes in GBM cell

Exosomal protein	Sample	Expression status	Note(result)	Target	Refs.
HMGB1	–	Up	HMGB1 has various functions depending on where it is found: as an extracellular protein, it downregulates SASH1, whereas as an exosomal protein, it upregulates SASH1	SASH1	[223]
IL-8, PDGFs, caveolin 1, and lysyl oxidase	Glioma patient	Up	During tumor progression, the exosomal pathway could be a possible target for induction of hypoxia-dependent intercellular signaling		[61]
L1CAM	Human T98G GBM cell line	Up	Cell movement, growth, and invasiveness are all increased	FGFR, FAK	[224]
STC1, STC2	Human malignant glioma U373MG cells	Up	Cell immigration is induced in a hypoxia-dependent way		[225]
EGFRvIII	Human astrocytoma (U373vIII)	Up	-	CD44, BSG, CD151, CD81 and CD82	[14]
VEGF-A	Human glioma cell lines (U87, U251)	Up	In vitro by disrupting expression of claudin-5 and occluding permeability of BBB could be increased; exosomes, which are generated by GBM in hypoxic conditions, could stay active and make BBB to be more permeable according to in vivo study	claudin-5 and occluding	[226]
CRYAB	U373 glioma cells	Up	cryAB are generated and released via exosomes by U373 glioma cells both the amount of cryAB in cell and released levels by exosomes in cells are remarkably elevated when induced by IL-1β and TNF-α		[227]
PTRF	Clinical glioma samples (tissue and serum)	Up	In vitro proliferation of cell and generation of exosomes are stimulated when PTRF are over expressed. In cancer tissue and exosomes separated from glioma patients, positive linked between grade of tumor and expression of PTRF have been shown	Cavin1	[228]
IL13Rα2, IL13QD	Glioma stem cells	Up	It has been proved that there is special binding between exosomes generated by tumor and IL13QD		[229]
CAV1	Glioblastoma U87 cells	Up	It seems that exosome uptake reliant on the presence of an intact ERK1/2-HSP27 complex. CAV1 had a negative impact on ERK1/2 phosphorylationduring exosome internalization	p-ERK1/2	[230]
CLIC1	GBM cell lines	Up	CLIC1 which are generated by GBM-derived CSCs or cell lines is released through exosomes is a protein in blood	GFP, FLAG-tagged	[231]
TrkB	GBM cell lines	Up	In regulation of GBM development and invasiveness has a vital role	YKL-40	[232]
N-glycoproteins	Human plasma	Up	in exosomes which isolated from sera of both healthy and patients with glioma 180 different N-glycoproteinswere enriched and recognized which correspond to 329 N-glycosylation sites	Glycopeptide	[233]
LOX, ADAMTS1, TSP1, VEGF	Human glioma cell line, U87MG	Up	Expression of various genes in recipient glioma cells are stimulated	KCNJ3	[234]

## Recent detection methods for exosomal biomarkers

Intracranial biopsies, magnetic resonance imaging (MRI), and computed tomography (CT) scans are the conventional methods for glioma diagnosis and prognosis [104, 105]. However, these methods fail to detect the precise molecular signatures of glioma progression and metabolic adaptation. Given these circumstances, the design and development of novel diagnostic tools to monitor the progression of glioma and its metabolic reprogramming are imminently required. These tools could be used to detect the biomarkers (tumor-associated proteins and microRNA) of glioma- derived exosomes [62]. Mutant isocitrate dehydrogenase 1, mutant epidermal growth factor receptor variant III (EGFRvIII), tumor-specific mRNA and microRNAs, and microRNA-21 are among the biomarkers carried by glioma- derived exosomes [106, 107].

Highly sensitive detection of glioma exosomes and their biomarkers are suggested to improve the accuracy of diagnosis and prognosis [61, 88, 108]. In this regard, surface proteins of glioma-derived exosomes can be deemed reliable diagnostic biomarkers for a thorough understanding of glioma progression and metabolic adaptation. Detection tools such as surface-enhanced Raman scattering (SERS), localized surface plasmon resonance (LSPR), atomic force microscopy (AFM) and other advanced technologies can measure these membrane markers [109–111]. These advanced methods are cost-effective, label-free, real-time, and highly sensitive.

LSPR is a biocompatible biosensing technique, which allows for highly sensitive detection of biomarkers. The altered dielectric property of surroundings in the functionalized sensing chip of LSPR, endows this technique with a high spatial resolution (26). These properties of LSPR lead to sensitive detection of single molecular interactions, such as antigen–antibody interactions. LSPR has recently been used for the detection of surface proteins (biomarkers) displayed on the exosomes derived from various tumors such as the glioma [9, 11, 112]. AFM is a versatile scanning probe microscope that has also been used for the detection biomarkers expressed on glioma-derived exosomes [9, 113, 114]. Detecting the adhesive forces between the functionalized probe tips and the sample, this technique provides nanoscale spatial resolution to measure single molecular interactions [115]. AFM is considered an amenable tool to analyze the biological samples, due to its ability for low damage imaging of soft samples in air and liquid such as exosomes [116]. SERS method has also been suggested for label-free exosome detection and exosome detection with SERS-tags from different sources including glioma [117–120]. SERS is among the nanomaterial-based optical biosensors, which is a powerful optical technique for biosensing and clinical diagnostic. Various other intriguing techniques have also been developed for rapid and sensitive exosome detection and discrimination. This growing field would bring about ultrasensitive detection tools for the exosome based diagnostics of cancers.

## Machine learning in the prediction of exosome based biomarker

The wealth of proteomic and genetic information, which is carried by circulating exosomes, presents an enormous opportunity for cancer diagnostics. However, the existing heterogeneity between patients and within a tumor itself could confound the analysis of exosomal biomarkers. Targeting a single biomarker could be influenced by many complex processes and may not directly map to a specific disease state that is universally true for all patients. To address this challenge, a panel of molecular biomarkers could be measured to get a better profile of cancer state. Machine learning algorithms and training sets of data could be used to make sense of the multiple molecular biomarkers within a profile. These tools would extract a set of optimized linear discriminators from an existing panel of biomarkers. Artificial intelligence (AI) based machine learning (ML) algorithms such as support vector machine (SVM) and decision tree model could also be used to identify exosome based potential biomarkers of various malignancies and predict the diagnostic and prognostic outcomes of pathological conditions [121–123]. In light of accumulated evidence, a combination of exosome analysis and deep learning promises a great-potential as a method for early stage cancer diagnosis [124].

## Peptide functionalization of exosomes for glioma therapy

Peptides have recently been conjugated to various nano delivery systems (NDS) such as exosome for targeted delivery of therapeutics into cancer cites and in vivo imaging and tracking. Peptide-based functionalization of exosomes has been extensively studied for cancer targeting as potential next generation biological tools. These exosomes show augmented ability to target specific receptors or mutant proteins, which are displayed on the surface of cancer cells [17]. Surface modification of exosomes could play a pivotal role in the adaptation of targeting ability against brain, breast, lung, liver, colon tumors, and heart diseases. It could also be used to understand theirs in vivo fate including their pharmacokinetics, uptake mechanisms, and bio-distribution. The surface of exosomes could be modified via various approaches. Physical approaches in exosome modification such as sonication, extrusion, and freeze–thaw can change the surface properties of exosomes via membrane rearrangements. On the other hand, biological approaches such as genetic and metabolic engineering of the source cells could express protein or cargo molecules of interest in secreted exosomes [125]. Lactadherin (LA), tetraspanins (CD63, CD81, CD9), glycosyl-phosphatidyl-inositol (GPI), and lysosome associated membrane protein-2b (Lamp-2b) are among the most specific exosomal membrane proteins, which are involved in the functionalization of exosome surface [125]. Furthermore, functionalization of peptides onto the exosome surfaces may bring about substantial advantages for selective target binding and therapeutic effects for brain tumor treatment [126]. In this regard, biofunctional peptide-modified exosomes, as novel drug delivery systems, have been previously developed and successfully demonstrated [127]. Various studies have also practiced the peptide functionalization of exosomes for glioma therapy [126, 128, 129]. Moreover, this approach could pave the way for the conjugation of exosomes with receptor-targeted peptides for cancer therapy and diagnosis. These properties unveil the huge potency of peptide functionalization of exosomes in future cancer treatment and diagnosis [17].

## Exosome mediated metabolic reprogramming and immunomodulatory effects

The importance of glioma-derived EVs have already been highlighted as message carriers and mediators of immune escape. These EVs have the capacity to reprogram tumor-infiltrating immune cells [130]. Zeng et al. reported that glioblastoma-derived EVs could induce proliferation, self-renewal, and colony formation. Therefore, they could facilitate the transformation of astrocytes via reprogramming of oncogenic metabolism and enhanced neoplastic growth of astrocytoma in a mouse allograft model. Induction of a shift in gene expression plays a crucial role in the reprogramming of astrocyte metabolism. This expression shift may be partly exerted via the EV-mediated transfer of full-length mRNAs encoding oxidative phosphorylation, ribosomal proteins, and glycolytic factors [131]. Moreover, glioma cell mediated immunosuppressive functions could be broadened and amplified via various bioactive cargoes of glioma-derived EVs. In vitro enrichment of glioblastoma-derived exosomes with inhibitory proteins could dramatically suppress immune cell activities. Azambuja et al. have demonstrated that internalization of glioblastoma-derived exosomes via macrophages makes them highly susceptible to reprogramming. M1 polarization to M2 was shown to be achieved by FasL-induced activation of the NF-κB pathway in macrophages. Moreover, blocking of NF-κB signaling has been shown to reverse the M2 phenotype to M1. Promoted glioblastoma progression has also been shown via glioblastoma-derived exosomes. The frequency of CD8 + T cells and M1 macrophages has been decreased following the injection of glioblastoma-derived exosomes, while the M2 cells have been increased in the spleen. Protection of immune cells, especially CD8 + T cells and M1 macrophages, from the effects of glioblastoma-derived exosomes has emerged as a potential therapeutic target for future glioblastoma immunotherapy [132]. It has been shown that co-incubation with glioblastoma-derived exosomes could trigger the acquisition of fibroblast shape by macrophages and expression of M2 markers, including Arginase-1, IL-10, CD206, and LAP. In contrast, M1 markers such as CD80, CD86, and INF-γ remain unchanged in comparison to controls with no glioblastoma-derived exosomes. In light of these observations, a more essential role could be predicted for glioblastoma-derived exosomes in metabolic reprogramming and the exertion of immunomodulatory effects.

## Other treatment strategies for Glioma

### Current approach and strategies for treatment of glioma

Three essential treatment of high-grade gliomas are maximal surgical resection, external beam radiation therapy, and chemotherapy. Many brain tumors with a multidisciplinary team, including neurosurgeons, radiologists, pathology, radiology oncology and neuronecology are surgically removed. Pre-operative imaging is a mainstay for safe and effective surgical resection. Eloquent cortex is now mapped with several different imaging techniques including functional MRI (fMRI) [133], magnetoencephalography (MEG) [134], navigated transcranial magnetic stimulation (nTMS) [135], and diffusion tensor imaging fiber tracking (DTI-FT) [136].

Although, low-grade gliomas were treated with external beam radiation. In addition, the standard care for treatment of high-grade gliomas includes temozolamide an oral cytotoxic DNA-alkylating chemotherapy. There are many limitations to the current chemotherapeutics used for glioma. Systemically delivered drugs usually do not reach high concentrations within the CNS and at the tumor site. In addition, they lead to significant systemic side effects such as myelosuppression. Novel drug delivery systems such as nanoparticles could improve the distribution of the agents directly to the brain tumor [137]. There are several approaches to direct CNS delivery including injection into the CSF or cyst cavity with an implanted reservoir, implantable controlled-release polymer systems into the surgical cavity, and catheter-based convection-enhanced delivery (CED). Nonetheless, direct CNS delivery is influenced by many factors including infusion rate, diffusion rate, and gradient, which is the influenced by the properties of the therapeutic, tumor, and interstitial space [138]. Moreover, developed research is in intra-tumoral gene therapy. Antiangiogenic strategies targeting VEGF are currently undergoing extensive research and use for treatment of glioma [137]. Further strategies for glioma therapy are immunotherapy strategies. Tumor-associated antigens are selectively expressed on tumor cells, but can also be found on normal cells that produce relatively weak immune response secondary to central tolerance [137, 139]. Nowadays, the treatment of gliomas will incorporate translational research efforts focusing on precision medicine, personalized care, and clinical trials. It seems that exosome therapy, as pointed out, offers a vast array of new technologies with many permutations for the treatment of glioma tumors.

### Monoclonal antibodies in Glioma immunotherapy

A single B-cell clone mediates the production of monoclonal antibodies (mAbs). The mAbs could specifically bind to a single specific epitope, which makes them amenable therapeutic and diagnostic moieties. Köhler and Milstein were the first to devise a method to produce mAbs, called hybridoma method [140, 141]. Using mAbs could improve the clinical outcomes and patient survival rates. Increased survival has mainly, been observed in both inflammatory and neoplastic diseases. The immune system recognizes the antigens displayed on cancer cells which would lead to eradication of pathogens, elimination of tumor cells, and helps to maintain homeostasis [142, 143]. However, some cancers such as GBM evade immune surveillance via overexpression of immune checkpoint ligands. To overcome this phenomenon, a number of monoclonal antibodies have been introduced to target and suppress these immune checkpoints [144, 145]. It has been reported that angiogenesis has pivotal role in supplying oxygen and nutrients to developing gliomas tumors. VEGF and endothelial, stromal, and tumor cells are the leading proangiogenic factors stimulating to stimulate vessel growth and tumor expansion. Bevacizumab and Aflibercept applied against VEGF [146, 147]. Developing novel strategies that enhance the ability of therapeutic antibodies crossing the BBB seems to be necessary. It has been suggested that the designing the conjugated nanoparticles with antineoplastic antibodies have high specificity and increase the focal levels of drugs. Since this approach requires the delivery of such nanoparticles into the tumor, conventional systemic administration of them would be associated with decreased adverse events [148]. Contemporary, conjugate drugs have become a new strategy in anticancer therapy. For example, decreased size of therapeutic antibodies to conjugate with nanoparticles is introduced as a novel approach to alleviate their delivery into CNS tumors, which are poorly accessible.

### The application of Bi-specific antibodies in Glioma immunotherapy

Bispecific antibodies (bsAb) have been reported to overcome the insufficiencies of the conventional treatments with mAbs. The main advantage of bispecific antibodies is the simultaneous binding possibility to two different antigens. Bispecific antibodies could target two different receptors on the same cell inducing some changes in cell signaling [149]. There are two main types of bispecific antibodies including IgG-like and non-IgG-like types[150]. Up to 60 bi-specific antibodies are under preclinical testing and more than 30 bispecific antibodies are undergoing clinical trials simultaneously. Approximately two-thirds of bispecific antibodies are devoted to cancer therapy [151]. However, bispecific antibodies have also been introduced for other diseases and abnormalities such as autoimmunity, infections, hemophilia, and Alzheimer's disease. The latest strategies to design bsAbs include phage display detection, antibody binding engineering, quaternary technology, knot-in-hole technology, ordinary light chain, CrossMAb technology, and also protein engineering. Every hybridoma cell expresses its own mouse monoclonal antibody specificity, thus the produced bsAb has two arms that are different and has their own specificities. Compared to normal antibodies, the bsAb produced by Quadroma has a longer half-life, higher solubility, and better stability. The disadvantage of the current method is linked to its low efficiency, due to the production of non-functional antibodies [151, 152]. In Knobs-into-holes (KiH) technique, the CH3 domain of an antibody is designed to enhance Fc hetero-dimerization. The design of bispecific antibodies exploiting the KiH method needs special care about the distance between the alpha carbons, the desired conformation, and the type of the amino acid. The antibodies obtained by this method are highly stability, form correct heterodimers, and can be purified by protein A column [153, 154]. Bispecific antibodies, can be developed by CrossMAb technology. They can be bi-, tri-, tetravalent and novel Fab-based antibody [155, 156]. We principally focus on the most recent advances in remedial bispecific antibodies for glioma disease. In the first phase clinical preliminaries, promising outcomes have been shown to utilize bispecific antibodies focusing on CD3 and a glioma antigen [157, 158]. Of note, CD8 + T-cell invasion is correlated with delayed endurance of recently analyzed GBM patients [159]. Furthermore, practically 50% of the T cells penetrating GBM were CD56 + T cells [160], and anti-CD3 × anti-GD2 bispecific antibody had the option to divert T-cell cytolytic action to a neuroblastoma target [161]. The lack of improvement in the results of GBM cannot be inferred to the stem cells of glioma (GSC), which are small subpopulations of the cells involved in the invasion of the tumor, recurrence, and tolerance to chemo or radiotherapy. The population of GSC is challenging to both empirically define and treat. GSCS is described by self-forming and differentiation capacity to modify GBM heterogeneity [162]. Multiple strategies for GST based therapy are currently under investigation at preclinical and different clinical stages [163]. Recent studies have presented evidences to support the general origin of the GBM stem cell, and provide an overview of the standard limitations for GBM treatment. BsAb and related innovations have shown potential in reinforcing the counter GSC impacts of latent immunotherapies. A bispecific counter acting agent against CD133 and EGFRvIII was exhibited to be profoundly cytotoxic against GSCs (however not NSCs). It also was more successful in drawing out OS in mice when contrasted with CD133 or EGFRvIII mAbs alone [164]. The expanded efficacy of bispecific antibodies may show more prominent anti-GSC impacts and diminish poisonousness compared to the monotherapies [165, 166].

### mRNA and DC vaccines for glioma cancer

Numerous studies are carried out to design vaccines for prevention of Glioma or its post-treatment recurrence. Vaccination with protein moieties mainly triggers the humoral immune response, while the fight against cancer needs both cellular and the humoral branches of the immune system. The pathogen-associated microbial pattern molecules, described as “danger molecules” such as lipopolysaccharides (LPS). These molecules are characterized by the release of interleukin 12 (IL-12) [167–169]. DCs are antigen-presenting cells that mediate immune reactions [170, 171].

**Fig. 2:**
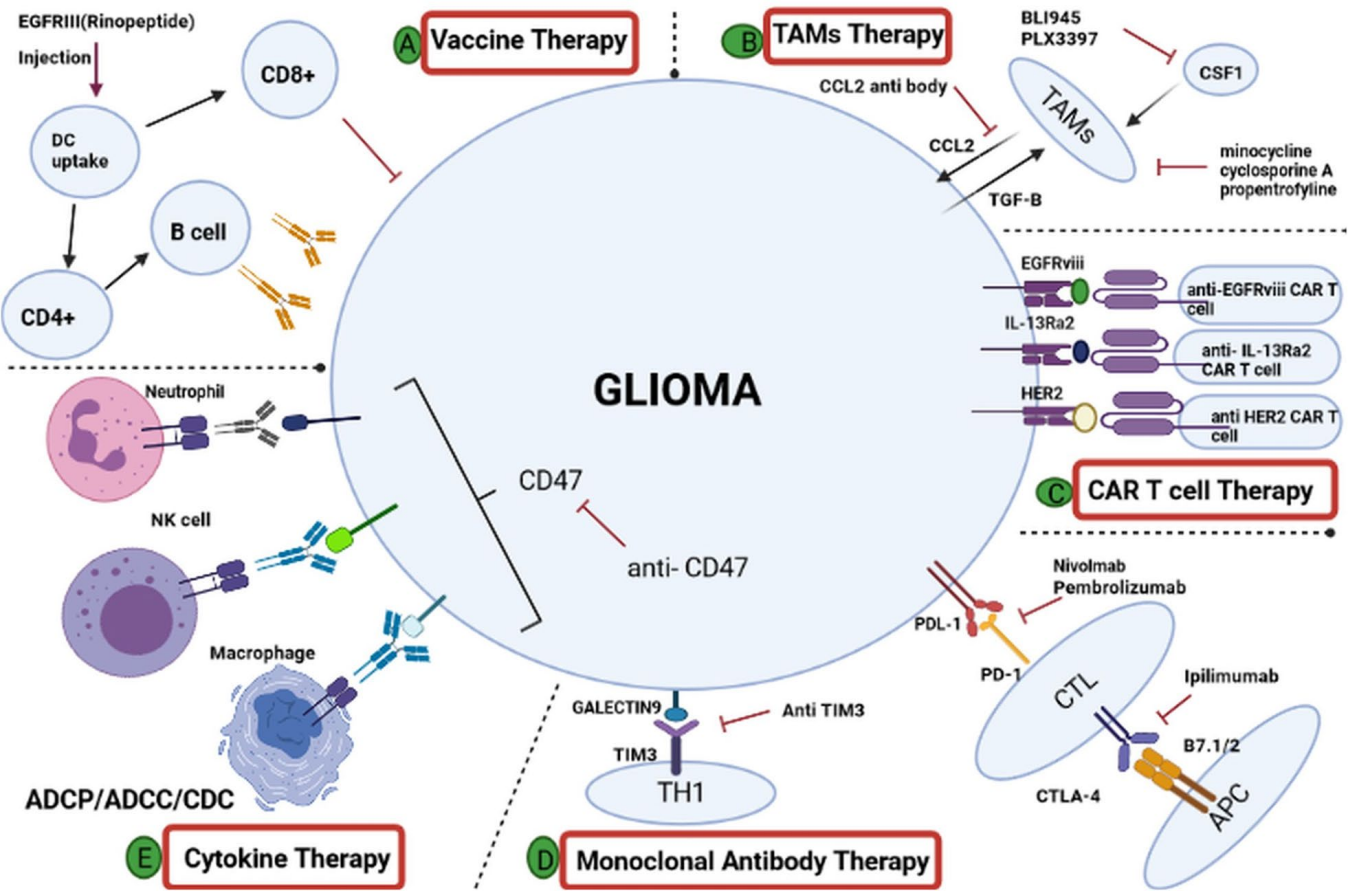
Novel therapeutic strategies for patients with glioma. **A** Vaccines for glioma treatment or prevention of recurrence. Different types of vaccines include peptide, dendritic cells, DNA, RNA, and viral vaccine vectors, which are in various phases of clinical trials. **B** Tumor-associated macrophages (TAMS) therapy resulted in neoangiogenesis and the invasive growth of GBMs. **C** Car T cell therapy. **D** Monoclonal antibody therapy. **E** Cytokine therapy modulating the tumor microenvironment

In contact with pathogen-associated microbial pattern molecules, DCs switch into a potent immune-stimulatory mode of action called “maturation”. This property distinguishes the “Audience” technology, from other DC-based cancer immunotherapy technologies [172]. Audience is cellular cancer immunotherapy, which uses dendritic cells against GBM. The recent phase II “GBM-Vax” trial has announced the Audencel to be clinically ineffective as assessed by progression-free and survival of all patients [172, 173]. Erhart et al. have also conducted a phase II Audencel trail. They have reported that although the Audencel seems to → stimulate the immune system, the outcomes of therapy are influenced by the state of the immune system [174] (Fig. 2). Novel strategies are imminently required for glioma treatments. In this regard, some studies have been devoted to the production of vaccines that target the Cytomegalovirus (CMV) protein pp65 in GBM. It has been reported that more than 90% of GBMs express the CMV proteins [175]. Since these proteins have specifically been detected in glioma cells and are absent in the surrounding normal brain, they could be deemed as tumor-specific immunotherapy targets [176]. CMV-specific DC vaccines could be employed against newly diagnosed GBMs. The pp65 CMV antigen has previously been targeted with autologous DC vaccination following standard of care resection and chemo-radiation [177]. Autologous DC vaccine production and administration have followed the same treatment schedule with TMZ adjuvant in all three trials [177]. Batich et al. have been used pp65-lysosomal-associated membrane protein (LAMP)-based mRNA DC vaccine and studied a median overall survival of 35 months, and progression-free survival of 31 months was demonstrated for this vaccine [178]. DNA or RNA based vaccines [179] (especially non-coding RNA) [180] [179] are new vaccine platforms that are under intense investigation. Various studies have indicated the ability of nucleic acid vaccines for the induction of immune response against different diseases. Several studies have shown that dysregulation of mir-137 is related to the aggressive progression of glioma [181, 182]. MiR-137 regulates the proliferation of the glioma cells via the Akt/mammalian target of rapamycin (mTOR) signaling pathway [183]. It has also has been reported that miR-137 inhibits cell growth by blocking the Wnt/b-catenin pathway and negative regulation of FOXK1 expression in glioma cells [184]. Wnt signaling as one of the key signaling pathway regulates human tumor growth and development, especially cell proliferation. The Wnt/b-catenin signaling pathway have been confirmed in several studies that mediates glioma growth [185].

## Conclusion

Glioma therapy based on the role of exosomes in the initiation and progression of cancer has recently been well stabilized. The rapid development of drug resistance in tumor cells has become the most significant challenge in cancer treatment. Hence, understanding the common molecular mechanisms, which are involved in drug resistance and the complex interaction among different components of the tumor microenvironment, could lead to success in therapeutic strategies. The primary studies were mainly focused on progressive cancerous cells. EVs, especially exosomes, are now classified as a part of a novel inter-cellular communication system. The exosome-based diagnostic is suggested as a non-invasive method to obtain a tissue sample without the need for surgery. Several exosomal molecular signatures have been used as a biomarker by detecting the oxygenation status and development of tumors. Exosomes could be detected in almost all body fluids and they could provide information about the parental cells that originally produced them. In light of these insights, exosomes have become a novel area of interest for researchers worldwide.

In this regard, we highlighted in this review the possible role of exosomes and exosomal microRNA in glioma cancer. We emphasized more on the key modulating molecules, clinical relevance to glioma, and associated signaling pathways. Finally, it was established that sharing exosome contents with a possible role in chemo-resistance could help to overcome the drug resistance. Although the isolation and purification of exosomes are yet to become standardized, large-scale use of exosomes in clinical trials is vitally necessary. Finally, by using cancer-derived exosomes in treating glioma, the complement analyses are needed to ensure they are free from angiogenic proteins that stimulate the initiation of angiogenesis and metastasis in glioma.

## Future directions

Several remarkable studies have revealed that exosome research is now expanding, an in-depth understanding of subcellular components and mechanisms involved in exosome formation and specific cell-targeting will bring light in their physiological activities. Currently, exosomes could be used as valuable diagnostic and prognostic biomarkers for their cell-lineage and state-specific contents, and possibilities as therapeutic vehicles for drug and gene delivery. Exosomes and their biologically active cargos may offer prognostic information in a range of diseases, such as chronic inflammation [186], cardiovascular and renal diseases [187, 188], neurodegenerative diseases [189], lipid metabolic diseases [190], and tumors. Exosomes potentially attract more attention as a programmable precision-guided drug delivery system. To this end, in addition to exosomes of native origins, researchers are trying to engineer exosomes for specific contents [191–193].

## Data Availability

The datasets used and/or analyzed during the current study are available from the corresponding author on reasonable request.

## Abbreviations

CNS: Central nervous system
GBM: Glioblastoma multiforme
BBB: Blood–brain barrier
IDH: Isocitrate dehydrogenase
TCF12: Transcription factor 12
PIK3R1: PI3K-regulatory subunit 1
NF1: Neurofibromatosis type 1
PDGFRA: Platelet Derived Growth Factor Receptor Alpha
H3K27me3: Trimethylation of histone H3 at lysine 27
ILVs: Intraluminal vesicles
TME: Tumor microenvironment
GSC: Glioma stem cells
VEGF: Vascular endothelial growth factor
TCGA: The cancer genome atlas
PTEN: Phosphatase and tensin homolog
PI3K: Phosphatidylinositol 3-kinase
BsAb: Bispecific antibodies (bsAb)
KiH: Knobs-into-holes
LPS: Lipopolysaccharides

## References

[1] Rock K, McArdle O, Forde P, Dunne M, Fitzpatrick D, O'Neill B (2012). A clinical review of treatment outcomes in glioblastoma multiforme—the validation in a non-trial population of the results of a randomised Phase III clinical trial: has a more radical approach improved survival?. Br J Radiol.

[2] Hanif F, Muzaffar K, Perveen K, Malhi SM, Simjee SU (2017). Glioblastoma multiforme: a review of its epidemiology and pathogenesis through clinical presentation and treatment. Asian Pac J Cancer Prev APJCP.

[3] Agnihotri S, Burrell KE, Wolf A, Jalali S, Hawkins C, Rutka JT (2013). Glioblastoma, a brief review of history, molecular genetics, animal models and novel therapeutic strategies. Arch Immunol Ther Exp.

[4] Dong X (2018). Current strategies for brain drug delivery. Theranostics.

[5] Karami Fath M, Karimfar N, Fazlollahpour Naghibi A, Shafa S, Ghasemi Shiran M, Ataei M (2022). Revisiting characteristics of oncogenic extrachromosomal DNA as mobile enhancers on neuroblastoma and glioma cancers. Cancer Cell Int.

[6] Harding CV, Heuser JE, Stahl PD (2013). Exosomes: looking back three decades and into the future. J Cell Biol.

[7] Maia J, Caja S, Strano Moraes MC, Couto N, Costa-Silva B (2018). Exosome-based cell-cell communication in the tumor microenvironment. Front Cell Dev Biol.

[8] Théry C, Witwer KW, Aikawa E, Alcaraz MJ, Anderson JD, Andriantsitohaina R (2018). Minimal information for studies of extracellular vesicles 2018 (MISEV2018): a position statement of the International Society for Extracellular Vesicles and update of the MISEV2014 guidelines. J Extracell Vesicles.

[9] Thakur A, Xu C, Li WK, Qiu G, He B, Ng S-P (2021). In vivo liquid biopsy for glioblastoma malignancy by the AFM and LSPR based sensing of exosomal CD44 and CD133 in a mouse model. Biosens Bioelectron.

[10] Thakur A, Qiu G, Xu C, Han X, Yang T, Ng S (2020). Label-free sensing of exosomal MCT1 and CD147 for tracking metabolic reprogramming and malignant progression in glioma. Sci Adv.

[11] Xu C, Thakur A, Li Z, Yang T, Zhao C, Li Y (2021). Determination of glioma cells’ malignancy and their response to TMZ via detecting exosomal BIGH3 by a TiO2-CTFE-AuNIs plasmonic biosensor. Chem Eng J.

[12] Humbert M, Taillé C, Mala L, Le Gros V, Just J, Molimard M (2018). Omalizumab effectiveness in patients with severe allergic asthma according to blood eosinophil count: the STELLAIR study. Eur Respir J.

[13] Ebrahimkhani S, Vafaee F, Hallal S, Wei H, Lee MYT, Young PE (2018). Deep sequencing of circulating exosomal microRNA allows non-invasive glioblastoma diagnosis. NPJ Precis Oncol.

[14] Choi D, Montermini L, Kim D-K, Meehan B, Roth FP, Rak J (2018). The impact of oncogenic EGFRvIII on the proteome of extracellular vesicles released from glioblastoma cells. Mol Cell Proteomics.

[15] Thakur A, Parra DC, Motallebnejad P, Brocchi M, Chen HJ (2022). Exosomes: small vesicles with big roles in cancer, vaccine development, and therapeutics. Bioact Mater.

[16] Zhang X, Sai B, Wang F, Wang L, Wang Y, Zheng L (2019). Hypoxic BMSC-derived exosomal miRNAs promote metastasis of lung cancer cells via STAT3-induced EMT. Mol Cancer.

[17] Gaurav I, Thakur A, Iyaswamy A, Wang X, Chen X, Yang Z (2021). Factors affecting extracellular vesicles based drug delivery systems. Molecules.

[18] Haney MJ, Klyachko NL, Zhao Y, Gupta R, Plotnikova EG, He Z (2015). Exosomes as drug delivery vehicles for Parkinson's disease therapy. J Control Release.

[19] Thakur A, Sidu RK, Zou H, Alam MK, Yang M, Lee Y (2020). Inhibition of glioma cells’ proliferation by doxorubicin-loaded exosomes via microfluidics. Int J Nanomed.

[20] Calistri NL, Kimmerling RJ, Malinowski SW, Touat M, Stevens MM, Olcum S (2018). Microfluidic active loading of single cells enables analysis of complex clinical specimens. Nat Commun.

[21] Casadó A, Sagristá ML, Mora M (2018). A novel microfluidic liposomal formulation for the delivery of the SN-38 camptothecin: characterization and in vitro assessment of its cytotoxic effect on two tumor cell lines. Int J Nanomed.

[22] Louis D, Perry A, von Reifenberger DAG, Figarella-Branger D, Cavenee WK, Ohgaki H, Wiestler OD, Kleihues P, Ellison DW (2016). The 2016 World Health Organization classification of tumors of the central nervous system: a summary. Acta Neuropathol.

[23] Ma Q, Long W, Xing C, Chu J, Luo M, Wang HY (2018). Cancer stem cells and immunosuppressive microenvironment in glioma. Front Immunol.

[24] Wood MD, Halfpenny AM, Moore SR (2019). Applications of molecular neuro-oncology-a review of diffuse glioma integrated diagnosis and emerging molecular entities. Diagn Pathol.

[25] De Carli E, Wang X, Puget S (2009). IDH1 and IDH2 mutations in gliomas. N Engl J Med.

[26] Sasaki M, Knobbe CB, Munger JC, Lind EF, Brenner D, Brüstle A (2012). IDH1 (R132H) mutation increases murine haematopoietic progenitors and alters epigenetics. Nature.

[27] Rouse C, Gittleman H, Ostrom QT, Kruchko C, Barnholtz-Sloan JS (2015). Years of potential life lost for brain and CNS tumors relative to other cancers in adults in the United States, 2010. Neuro Oncol.

[28] Watson LA, Goldberg H, Bérubé NG (2015). Emerging roles of ATRX in cancer. Epigenomics.

[29] Chinot OL, Wick W, Mason W, Henriksson R, Saran F, Nishikawa R (2014). Bevacizumab plus radiotherapy–temozolomide for newly diagnosed glioblastoma. N Engl J Med.

[30] Jenkins RB, Blair H, Ballman KV, Giannini C, Arusell RM, Law M (2006). A t (1; 19)(q10; p10) mediates the combined deletions of 1p and 19q and predicts a better prognosis of patients with oligodendroglioma. Can Res.

[31] Stern JL, Theodorescu D, Vogelstein B, Papadopoulos N, Cech TR (2015). Mutation of the TERT promoter, switch to active chromatin, and monoallelic TERT expression in multiple cancers. Genes Dev.

[32] Bettegowda C, Agrawal N, Jiao Y, Sausen M, Wood LD, Hruban RH (2011). Mutations in CIC and FUBP1 contribute to human oligodendroglioma. Science.

[33] Alentorn A, Dehais C, Ducray F, Carpentier C, Mokhtari K, Figarella-Branger D (2015). Allelic loss of 9p21. 3 is a prognostic factor in 1p/19q codeleted anaplastic gliomas. Neurology.

[34] Arita H, Narita Y, Fukushima S, Tateishi K, Matsushita Y, Yoshida A (2013). Upregulating mutations in the TERT promoter commonly occur in adult malignant gliomas and are strongly associated with total 1p19q loss. Acta Neuropathol.

[35] Aldape K, Zadeh G, Mansouri S, Reifenberger G, von Deimling A (2015). Glioblastoma: pathology, molecular mechanisms and markers. Acta Neuropathol.

[36] Louis D, Ohgaki H, Wiestler O, Cavenee W, Ellison D, Figarella-Branger D, et al. World Health Organization classification of tumours of the central nervous system. Revised. Lyon: IARC. 2016. 10.1007/s00401-016-1545-1 27157931

[37] Kleinschmidt-DeMasters BK, Aisner DL, Birks DK, Foreman NK (2013). Epithelioid GBMs show a high percentage of BRAF V600E mutation. Am J Surg Pathol.

[38] Parker M, Mohankumar KM, Punchihewa C, Weinlich R, Dalton JD, Li Y (2014). C11orf95–RELA fusions drive oncogenic NF-κB signalling in ependymoma. Nature.

[39] Reifenberger G, Wirsching H-G, Knobbe-Thomsen CB, Weller M (2017). Advances in the molecular genetics of gliomas—implications for classification and therapy. Nat Rev Clin Oncol.

[40] Jones DT, Hutter B, Jäger N, Korshunov A, Kool M, Warnatz H-J (2013). Recurrent somatic alterations of FGFR1 and NTRK2 in pilocytic astrocytoma. Nat Genet.

[41] Bender S, Tang Y, Lindroth AM, Hovestadt V, Jones DT, Kool M (2013). Reduced H3K27me3 and DNA hypomethylation are major drivers of gene expression in K27M mutant pediatric high-grade gliomas. Cancer Cell.

[42] Lewis PW, Müller MM, Koletsky MS, Cordero F, Lin S, Banaszynski LA (2013). Inhibition of PRC2 activity by a gain-of-function H3 mutation found in pediatric glioblastoma. Science.

[43] Schwartzentruber J, Korshunov A, Liu X-Y, Jones DT, Pfaff E, Jacob K (2012). Driver mutations in histone H3. 3 and chromatin remodelling genes in paediatric glioblastoma. Nature..

[44] Wu G, Broniscer A, McEachron TA, Lu C, Paugh BS, Becksfort J (2012). Somatic histone H3 alterations in pediatric diffuse intrinsic pontine gliomas and non-brainstem glioblastomas. Nat Genet.

[45] Malmström A, Grønberg BH, Marosi C, Stupp R, Frappaz D, Schultz H (2012). Temozolomide versus standard 6-week radiotherapy versus hypofractionated radiotherapy in patients older than 60 years with glioblastoma: the Nordic randomised, phase 3 trial. Lancet Oncol.

[46] Wick W, Platten M, Meisner C, Felsberg J, Tabatabai G, Simon M (2012). NOA-08 Study Group of Neuro-oncology Working Group (NOA) of German Cancer Society Temozolomide chemotherapy alone versus radiotherapy alone for malignant astrocytoma in the elderly: the NOA-08 randomised, phase 3 trial. Lancet Oncol.

[47] Wick W, Meisner C, Hentschel B, Platten M, Schilling A, Wiestler B (2013). Prognostic or predictive value of MGMT promoter methylation in gliomas depends on IDH1 mutation. Neurology.

[48] Masui K, Mischel PS, Reifenberger G (2016). Molecular classification of gliomas. Handb Clin Neurol.

[49] Wick W, Weller M, Van Den Bent M, Sanson M, Weiler M, Von Deimling A (2014). MGMT testing—the challenges for biomarker-based glioma treatment. Nat Rev Neurol.

[50] Hu N, Richards R, Jensen R (2016). Role of chromosomal 1p/19q co-deletion on the prognosis of oligodendrogliomas: a systematic review and meta-analysis. Interdiscip Neurosurg.

[51] Vlassov AV, Magdaleno S, Setterquist R, Conrad R (2012). Exosomes: current knowledge of their composition, biological functions, and diagnostic and therapeutic potentials. Biochimica et Biophysica Acta General Subjects.

[52] Roma-Rodrigues C, Fernandes AR, Baptista PV (2014). Exosome in tumour microenvironment: overview of the crosstalk between normal and cancer cells. BioMed Res Int.

[53] Skotland T, Sandvig K, Llorente A (2017). Lipids in exosomes: current knowledge and the way forward. Prog Lipid Res.

[54] Chitra R, KB H. The Origin and Functions of Exosomes in Cancer. Frontiers in oncology. 2018;8(66). 10.3389/fonc.2018.00066 PMC5869252 29616188

[55] Kalluri R, LeBleu VS (2020). The biology, function, and biomedical applications of exosomes. Science.

[56] Rana S, Malinowska K, Zöller M (2013). Exosomal tumor microRNA modulates premetastatic organ cells. Neoplasia.

[57] De Toro J, Herschlik L, Waldner C, Mongini C (2015). Emerging roles of exosomes in normal and pathological conditions: new insights for diagnosis and therapeutic applications. Front Immunol.

[58] Eyvazi S, Hejazi MS, Kahroba H, Abasi M, Zamiri RE, Tarhriz V (2019). CDK9 as an appealing target for therapeutic interventions. Curr Drug Targets.

[59] Lapointe S, Perry A, Butowski NA (2018). Primary brain tumours in adults. Lancet.

[60] Sun Z, Wang L, Zhou Y, Dong L, Ma W, Lv L (2019). Glioblastoma stem cell-derived exosomes enhance stemness and tumorigenicity of glioma cells by transferring Notch1 protein. Cell Mol Neurobiol.

[61] Kucharzewska P, Christianson HC, Welch JE, Svensson KJ, Fredlund E, Ringnér M (2013). Exosomes reflect the hypoxic status of glioma cells and mediate hypoxia-dependent activation of vascular cells during tumor development. Proc Natl Acad Sci.

[62] Skog J, Würdinger T, Van Rijn S, Meijer DH, Gainche L, Curry WT (2008). Glioblastoma microvesicles transport RNA and proteins that promote tumour growth and provide diagnostic biomarkers. Nat Cell Biol.

[63] Chen J, Hou C, Wang P, Yang Y, Zhou D (2019). Grade II/III glioma microenvironment mining and its prognostic merit. World Neurosurgery.

[64] Ratajczak J, Wysoczynski M, Hayek F, Janowska-Wieczorek A, Ratajczak M (2006). Membrane-derived microvesicles: important and underappreciated mediators of cell-to-cell communication. Leukemia.

[65] Giusti I, Delle Monache S, Di Francesco M, Sanità P, D’Ascenzo S, Gravina GL (2016). From glioblastoma to endothelial cells through extracellular vesicles: messages for angiogenesis. Tumor Biol.

[66] Sun X, Ma X, Wang J, Zhao Y, Wang Y, Bihl JC (2017). Glioma stem cells-derived exosomes promote the angiogenic ability of endothelial cells through miR-21/VEGF signal. Oncotarget.

[67] Yue X, Lan F, Xia T (2019). Hypoxic glioma cell-secreted exosomal miR-301a activates Wnt/β-catenin signaling and promotes radiation resistance by targeting TCEAL7. Mol Ther.

[68] Zeng A, Wei Z, Yan W, Yin J, Huang X, Zhou X (2018). Exosomal transfer of miR-151a enhances chemosensitivity to temozolomide in drug-resistant glioblastoma. Cancer Lett.

[69] Wu X, Wang Y, Yu T, Nie E, Hu Q, Wu W (2017). Blocking MIR155HG/miR-155 axis inhibits mesenchymal transition in glioma. Neuro Oncol.

[70] Prados MD, Chang SM, Butowski N, DeBoer R, Parvataneni R, Carliner H (2009). Phase II study of erlotinib plus temozolomide during and after radiation therapy in patients with newly diagnosed glioblastoma multiforme or gliosarcoma. J Clin Oncol.

[71] Yin J, Zeng A, Zhang Z, Shi Z, Yan W, You Y (2019). Exosomal transfer of miR-1238 contributes to temozolomide-resistance in glioblastoma. EBioMedicine.

[72] Shi L, Cheng Z, Zhang J, Li R, Zhao P, Fu Z (2008). hsa-mir-181a and hsa-mir-181b function as tumor suppressors in human glioma cells. Brain Res.

[73] Thuringer D, Chanteloup G, Boucher J, Pernet N, Boudesco C, Jego G (2017). Modulation of the inwardly rectifying potassium channel Kir4. 1 by the pro-invasive miR-5096 in glioblastoma cells. Oncotarget.

[74] Wong H-KA, El Fatimy R, Onodera C, Wei Z, Yi M, Mohan A (2015). The Cancer Genome Atlas analysis predicts microRNA for targeting cancer growth and vascularization in glioblastoma. Mol Therapy.

[75] Attarha S, Roy A, Westermark B, Tchougounova E (2017). Mast cells modulate proliferation, migration and stemness of glioma cells through downregulation of GSK3β expression and inhibition of STAT3 activation. Cell Signal.

[76] Zhu Y, Zhang X, Wang L, Ji Z, Xie M, Zhou X (2017). Loss of SH3GL2 promotes the migration and invasion behaviours of glioblastoma cells through activating the STAT3/MMP2 signalling. J Cell Mol Med.

[77] Cai Q, Zhu A, Gong L (2018). Exosomes of glioma cells deliver miR-148a to promote proliferation and metastasis of glioblastoma via targeting CADM1. Bull Cancer.

[78] Guo X, Qiu W, Wang J, Liu Q, Qian M, Wang S (2019). Glioma exosomes mediate the expansion and function of myeloid-derived suppressor cells through microRNA-29a/Hbp1 and microRNA-92a/Prkar1a pathways. Int J Cancer.

[79] Lu Z, Liu M, Stribinskis V, Klinge C, Ramos K, Colburn N (2008). MicroRNA-21 promotes cell transformation by targeting the programmed cell death 4 gene. Oncogene.

[80] Yang CH, Yue J, Pfeffer SR, Fan M, Paulus E, Hosni-Ahmed A (2014). MicroRNA-21 promotes glioblastoma tumorigenesis by down-regulating insulin-like growth factor-binding protein-3 (IGFBP3). J Biol Chem.

[81] Belter A, Rolle K, Piwecka M, Fedoruk-Wyszomirska A, Naskręt-Barciszewska MZ, Barciszewski J (2016). Inhibition of miR-21 in glioma cells using catalytic nucleic acids. Sci Rep.

[82] Sicard F, Gayral M, Lulka H, Buscail L, Cordelier P (2013). Targeting miR-21 for the therapy of pancreatic cancer. Mol Ther.

[83] Devulapally R, Sekar NM, Sekar TV, Foygel K, Massoud TF, Willmann JrK (2015). Polymer nanoparticles mediated codelivery of antimiR-10b and antimiR-21 for achieving triple negative breast cancer therapy. ACS Nano.

[84] Monfared H, Jahangard Y, Nikkhah M, Mirnajafi-Zadeh J, Mowla SJ (2019). Potential therapeutic effects of exosomes packed with a miR-21-sponge construct in a rat model of glioblastoma. Front Oncol.

[85] Chang L, Lei X, Qin Y, Zhang X, Jin H, Wang C (2015). MicroRNA-133b inhibits cell migration and invasion by targeting matrix metalloproteinase 14 in glioblastoma. Oncol Lett.

[86] Lan F, Qing Q, Pan Q, Hu M, Yu H, Yue X (2018). Serum exosomal miR-301a as a potential diagnostic and prognostic biomarker for human glioma. Cell Oncol.

[87] Manterola L, Guruceaga E, Pérez-Larraya JG, González-Huarriz M, Jauregui P, Tejada S (2014). A small noncoding RNA signature found in exosomes of GBM patient serum as a diagnostic tool. Neuro Oncol.

[88] Shi R, Wang P-Y, Li X-Y, Chen J-X, Li Y, Zhang X-Z (2015). Exosomal levels of miRNA-21 from cerebrospinal fluids associated with poor prognosis and tumor recurrence of glioma patients. Oncotarget.

[89] Chao MP, Weissman IL, Majeti R (2012). The CD47–SIRPα pathway in cancer immune evasion and potential therapeutic implications. Curr Opin Immunol.

[90] Koh E, Lee EJ, Nam G-H, Hong Y, Cho E, Yang Y (2017). Exosome-SIRPα, a CD47 blockade increases cancer cell phagocytosis. Biomaterials.

[91] Chalhoub N, Baker SJ (2009). PTEN and the PI3-kinase pathway in cancer. Annu Rev Pathol.

[92] Vazquez F, Matsuoka S, Sellers WR, Yanagida T, Ueda M, Devreotes PN (2006). Tumor suppressor PTEN acts through dynamic interaction with the plasma membrane. Proc Natl Acad Sci.

[93] Lachyankar MB, Sultana N, Schonhoff CM, Mitra P, Poluha W, Lambert S (2000). A role for nuclear PTEN in neuronal differentiation. J Neurosci.

[94] Ginn-Pease ME, Eng C (2003). Increased nuclear phosphatase and tensin homologue deleted on chromosome 10 is associated with G0–G1 in MCF-7 cells. Can Res.

[95] Gil A, Andrés-Pons A, Fernández E, Valiente M, Torres J, Cervera J (2006). Nuclear localization of PTEN by a Ran-dependent mechanism enhances apoptosis: Involvement of an N-terminal nuclear localization domain and multiple nuclear exclusion motifs. Mol Biol Cell.

[96] Chang C-J, Mulholland DJ, Valamehr B, Mosessian S, Sellers WR, Wu H (2008). PTEN nuclear localization is regulated by oxidative stress and mediates p53-dependent tumor suppression. Mol Cell Biol.

[97] Song MS, Carracedo A, Salmena L, Song SJ, Egia A, Malumbres M (2011). Nuclear PTEN regulates the APC-CDH1 tumor-suppressive complex in a phosphatase-independent manner. Cell.

[98] Gimm O, Attie-Bitach T, Lees JA, Vekemans M, Eng C (2000). Expression of the PTEN tumour suppressor protein during human development. Hum Mol Genet.

[99] Perren A, Komminoth P, Saremaslani P, Matter C, Feurer S, Lees JA (2000). Mutation and expression analyses reveal differential subcellular compartmentalization of PTEN in endocrine pancreatic tumors compared to normal islet cells. Am J Pathol.

[100] Trotman LC, Wang X, Alimonti A, Chen Z, Teruya-Feldstein J, Yang H (2007). Ubiquitination regulates PTEN nuclear import and tumor suppression. Cell.

[101] Montemurro N (2020). Glioblastoma multiforme and genetic mutations: the issue is not over yet. An overview of the current literature. J Neurol Surg A Cent Eur Neurosurg.

[102] Putz U, Howitt J, Doan A, Goh C-P, Low L-H, Silke J (2012). The tumor suppressor PTEN is exported in exosomes and has phosphatase activity in recipient cells. Science Signal.

[103] Quezada C, Torres Á, Niechi I, Uribe D, Contreras-Duarte S, Toledo F (2018). Role of extracellular vesicles in glioma progression. Mol Aspects Med.

[104] Xu X, Yadav NN, Knutsson L, Hua J, Kalyani R, Hall E (2015). Dynamic glucose-enhanced (DGE) MRI: translation to human scanning and first results in glioma patients. Tomography.

[105] Hochberg FH, Pruitt A (1980). Assumptions in the radiotherapy of glioblastoma. Neurology.

[106] D’Asti E, Chennakrishnaiah S, Lee TH, Rak J (2016). Extracellular vesicles in brain tumor progression. Cell Mol Neurobiol.

[107] Chistiakov DA, Chekhonin VP (2014). Extracellular vesicles shed by glioma cells: pathogenic role and clinical value. Tumor Biol.

[108] Chandran VI, Welinder C, Månsson A-S, Offer S, Freyhult E, Pernemalm M (2019). Ultrasensitive immunoprofiling of plasma extracellular vesicles identifies syndecan-1 as a potential tool for minimally invasive diagnosis of glioma. Clin Cancer Res.

[109] Li J, Li Y, Li P, Zhang Y, Du L, Wang Y (2022). Exosome detection via surface-enhanced Raman spectroscopy for cancer diagnosis. Acta Biomater.

[110] Rupert DL, Claudio V, Lässer C, Bally M (2017). Methods for the physical characterization and quantification of extracellular vesicles in biological samples. Biochimica et Biophysica Acta (BBA)-General Subjects.

[111] Shao B, Xiao Z (2020). Recent achievements in exosomal biomarkers detection by nanomaterials-based optical biosensors—A review. Anal Chim Acta.

[112] Thakur A, Qiu G, Ng S-P, Guan J, Yue J, Lee Y (2017). Direct detection of two different tumor-derived extracellular vesicles by SAM-AuNIs LSPR biosensor. Biosens Bioelectron.

[113] Sharma S, Das K, Woo J, Gimzewski JK (2014). Nanofilaments on glioblastoma exosomes revealed by peak force microscopy. J R Soc Interface.

[114] Sharma S, LeClaire M, Gimzewski JK (2018). Ascent of atomic force microscopy as a nanoanalytical tool for exosomes and other extracellular vesicles. Nanotechnology.

[115] Hinterdorfer P, Baumgartner W, Gruber HJ, Schilcher K, Schindler H (1996). Detection and localization of individual antibody-antigen recognition events by atomic force microscopy. Proc Natl Acad Sci.

[116] Parisse P, Rago I, Ulloa Severino L, Perissinotto F, Ambrosetti E, Paoletti P (2017). Atomic force microscopy analysis of extracellular vesicles. Eur Biophys J.

[117] Ferreira N, Marques A, Águas H, Bandarenka H, Martins R, Bodo C (2019). Label-free nanosensing platform for breast cancer exosome profiling. ACS Sensors.

[118] Pang Y, Shi J, Yang X, Wang C, Sun Z, Xiao R (2020). Personalized detection of circling exosomal PD-L1 based on Fe3O4@ TiO2 isolation and SERS immunoassay. Biosens Bioelectron.

[119] Li J, Wang C, Yao Y, Zhu Y, Yan C, Zhuge Q (2020). Label-free discrimination of glioma brain tumors in different stages by surface enhanced Raman scattering. Talanta.

[120] Jalali M, Hosseini II, AbdelFatah T, Montermini L, Hogiu SW, Rak J (2021). Plasmonic nanobowtiefluidic device for sensitive detection of glioma extracellular vesicles by Raman spectrometry. Lab Chip.

[121] Thakur A, Mishra PA, Panda B, Sweta K, Majhi B (2020). Detection of disease-specific parent cells via distinct population of nano-vesicles by machine learning. Curr Pharm Des.

[122] Thakur A, Mishra PA, Panda B, Rodríguez CSD, Gaurav I, Majhi B (2020). Application of artificial intelligence in pharmaceutical and biomedical studies. Curr Pharm Des.

[123] Giangreco N, Lebreton G, Restaino S, Farr M, Colombo PC, Zorn E (2019). Exosome proteomics and machine learning identify novel biomarkers of primary graft dysfunction. J Heart Lung Transplant.

[124] Shin H, Oh S, Hong S, Kang M, Kang D, Ji Y-g (2020). Early-stage lung cancer diagnosis by deep learning-based spectroscopic analysis of circulating exosomes. ACS Nano.

[125] Salunkhe S, Dheeraj Basak M, Chitkara D, Mittal A (2020). Surface functionalization of exosomes for target-specific delivery and in vivo imaging & tracking: strategies and significance. J Control Release.

[126] Ye Z, Zhang T, He W, Jin H, Liu C, Yang Z (2018). Methotrexate-loaded extracellular vesicles functionalized with therapeutic and targeted peptides for the treatment of glioblastoma multiforme. ACS Appl Mater Interfaces.

[127] Nakase I (2021). Biofunctional peptide-modified extracellular vesicles enable effective intracellular delivery via the induction of macropinocytosis. Processes.

[128] Jia G, Han Y, An Y, Ding Y, He C, Wang X (2018). NRP-1 targeted and cargo-loaded exosomes facilitate simultaneous imaging and therapy of glioma in vitro and in vivo. Biomaterials.

[129] Kim G, Kim M, Lee Y, Byun JW, Hwang DW, Lee M (2020). Systemic delivery of microRNA-21 antisense oligonucleotides to the brain using T7-peptide decorated exosomes. J Control Release.

[130] Lucero R, Zappulli V, Sammarco A, Murillo OD, Cheah PS, Srinivasan S (2020). Glioma-derived miRNA-containing extracellular vesicles induce angiogenesis by reprogramming brain endothelial cells. Cell Rep.

[131] Zeng A, Wei Z, Rabinovsky R, Jun HJ, El Fatimy R, Deforzh E (2020). Glioblastoma-derived extracellular vesicles facilitate transformation of astrocytes via reprogramming oncogenic metabolism. iScience.

[132] Azambuja JH, Ludwig N, Yerneni S, Rao A, Braganhol E, Whiteside TL (2020). Molecular profiles and immunomodulatory activities of glioblastoma-derived exosomes. Neuro-Oncology Advances.

[133] Ulmer JL, Hacein-Bey L, Mathews VP, Mueller WM, DeYoe EA, Prost RW (2004). Lesion-induced pseudo-dominance at functional magnetic resonance imaging: implications for preoperative assessments. Neurosurgery.

[134] Ganslandt O, Buchfelder M, Hastreiter P, Grummich P, Fahlbusch R, Nimsky C (2004). Magnetic source imaging supports clinical decision making in glioma patients. Clin Neurol Neurosurg.

[135] Ottenhausen M, Krieg SM, Meyer B, Ringel F (2015). Functional preoperative and intraoperative mapping and monitoring: increasing safety and efficacy in glioma surgery. Neurosurg Focus.

[136] Berman JI, Berger MS, Mukherjee P, Henry RG (2004). Diffusion-tensor imaging—guided tracking of fibers of the pyramidal tract combined with intraoperative cortical stimulation mapping in patients with gliomas. J Neurosurg.

[137] Bush NAO, Chang SM, Berger MS (2017). Current and future strategies for treatment of glioma. Neurosurg Rev.

[138] Vogelbaum MA, Aghi MK (2015). Convection-enhanced delivery for the treatment of glioblastoma. Neuro-oncology.

[139] Phuphanich S, Wheeler CJ, Rudnick JD, Mazer M, Wang H, Nuno MA (2013). Phase I trial of a multi-epitope-pulsed dendritic cell vaccine for patients with newly diagnosed glioblastoma. Cancer Immunol Immunother.

[140] Lipman NS, Jackson LR, Trudel LJ, Weis-Garcia F (2005). Monoclonal versus polyclonal antibodies: distinguishing characteristics, applications, and information resources. ILAR J.

[141] Eyvazi S, Kazemi B, Dastmalchi S, Bandehpour M (2018). Involvement of CD24 in multiple cancer related pathways makes it an interesting new target for cancer therapy. Curr Cancer Drug Targets.

[142] Pardoll DM (2012). The blockade of immune checkpoints in cancer immunotherapy. Nat Rev Cancer.

[143] Hung AL, Garzon-Muvdi T, Lim M (2017). Biomarkers and immunotherapeutic targets in glioblastoma. World Neurosurg.

[144] Pourzardosht N, Hashemi ZS, Mard-Soltani M, Jahangiri A, Rahbar MR, Zakeri A (2020). Liothyronine could block the programmed death-ligand 1 (PDL1) activity: an e-Pharmacophore modeling and virtual screening study. J Recept Signal Transduct.

[145] Ramezani A, Zakeri A, Mard-Soltani M, Mohammadian A, Hashemi ZS, Mohammadpour H (2020). Structure based screening for inhibitory therapeutics of CTLA-4 unveiled new insights about biology of ACTH. Int J Pept Res Ther.

[146] Reardon DA, Conrad CA, Cloughesy T, Prados MD, Friedman HS, Aldape KD (2012). Phase I study of AEE788, a novel multitarget inhibitor of ErbB-and VEGF-receptor-family tyrosine kinases, in recurrent glioblastoma patients. Cancer Chemother Pharmacol.

[147] Batchelor TT, Mulholland P, Neyns B, Nabors LB, Campone M, Wick A (2013). Phase III randomized trial comparing the efficacy of cediranib as monotherapy, and in combination with lomustine, versus lomustine alone in patients with recurrent glioblastoma. J Clin Oncol.

[148] Hernández-Pedro NY, Rangel-López E, Vargas Félix G, Pineda B, Sotelo J (2013). An update in the use of antibodies to treat glioblastoma multiforme. Autoimmune Dis.

[149] Zhang X, Yang Y, Fan D, Xiong D (2017). The development of bispecific antibodies and their applications in tumor immune escape. Exp Hematol Oncol.

[150] Przepiorka D, Ko C-W, Deisseroth A, Yancey CL, Candau-Chacon R, Chiu H-J (2015). FDA approval: blinatumomab. Clin Cancer Res.

[151] Sedykh SE, Prinz VV, Buneva VN, Nevinsky GA (2018). Bispecific antibodies: design, therapy, perspectives. Drug Des Dev Ther.

[152] Spiess C, Zhai Q, Carter PJ (2015). Alternative molecular formats and therapeutic applications for bispecific antibodies. Mol Immunol.

[153] Ridgway JB, Presta LG, Carter P (1996). ‘Knobs-into-holes’ engineering of antibody CH3 domains for heavy chain heterodimerization. Protein Eng Des Sel.

[154] Yang Y, Guo R, Chen Q, Liu Y, Zhang P, Zhang Z (2018). A novel bispecific antibody fusion protein co-targeting EGFR and CD47 with enhanced therapeutic index. Biotech Lett.

[155] Klein C, Schaefer W, Regula JT (2016). The use of CrossMAb technology for the generation of bi-and multispecific antibodies. MAbs.

[156] Labrijn AF, Janmaat ML, Reichert JM, Parren PW (2019). Bispecific antibodies: a mechanistic review of the pipeline. Nat Rev Drug Discovery.

[157] Hishii M, Nitta T, Ebato M, Okumura K, Sato K (1994). Targeting therapy for glioma by LAK cells coupled with bispecific antibodies. J Clin Neurosci.

[158] Dillman RO, Duma CM, Schiltz PM, DePriest C, Ellis RA, Okamoto K (2004). Intracavitary placement of autologous lymphokine-activated killer (LAK) cells after resection of recurrent glioblastoma. J Immunother.

[159] Yang I, Tihan T, Han SJ, Wrensch MR, Wiencke J, Sughrue ME (2010). CD8+ T-cell infiltrate in newly diagnosed glioblastoma is associated with long-term survival. J Clin Neurosci.

[160] Waziri A, Killory B, Ogden AT, Canoll P, Anderson RC, Kent SC (2008). Preferential in situ CD4+ CD56+ T cell activation and expansion within human glioblastoma. J Immunol.

[161] Yankelevich M, Kondadasula SV, Thakur A, Buck S, Cheung NKV, Lum LG (2012). Anti-CD3× anti-GD2 bispecific antibody redirects T-cell cytolytic activity to neuroblastoma targets. Pediatr Blood Cancer.

[162] Lathia JD, Mack SC, Mulkearns-Hubert EE, Valentim CL, Rich JN (2015). Cancer stem cells in glioblastoma. Genes Dev.

[163] Gimple RC, Bhargava S, Dixit D, Rich JN (2019). Glioblastoma stem cells: lessons from the tumor hierarchy in a lethal cancer. Genes Dev.

[164] Epidermal growth factor receptor variant III (EGFRvIII); prominin 1 (PROM1; CD133). Science-Business eXchange, 2014;7(6):172.

[165] Patel R, Baker SS, Liu W, Desai S, Alkhouri R, Kozielski R (2012). Effect of dietary advanced glycation end products on mouse liver. PLoS ONE.

[166] Li F, Lv B, Liu Y, Hua T, Han J, Sun C (2018). Blocking the CD47-SIRPα axis by delivery of anti-CD47 antibody induces antitumor effects in glioma and glioma stem cells. Oncoimmunology.

[167] Medzhitov R, Janeway CA (2002). Decoding the patterns of self and nonself by the innate immune system. Science.

[168] Luger R, Valookaran S, Knapp N, Vizzardelli C, Dohnal AM, Felzmann T (2013). Toll-like receptor 4 engagement drives differentiation of human and murine dendritic cells from a pro-into an anti-inflammatory mode. PLoS ONE.

[169] Payandeh Z, Yarahmadi M, Nariman-Saleh-Fam Z, Tarhriz V, Islami M, Aghdam AM (2019). Immune therapy of melanoma: overview of therapeutic vaccines. J Cell Physiol.

[170] Steinman RM, Cohn ZA (1974). Identification of a novel cell type in peripheral lymphoid organs of mice II. Functional properties in vitro. J Exp Med.

[171] Steinman RM, Cohn ZA (1973). Identification of a novel cell type in peripheral lymphoid organs of micemorphology, quantitation tissue distribution. J Exp Med.

[172] Felzmann T, Hüttner KG, Breuer SK, Wimmer D, Ressmann G, Wagner D (2005). Semi-mature IL-12 secreting dendritic cells present exogenous antigen to trigger cytolytic immune responses. Cancer Immunol Immunother.

[173] Hüttner KG, Breuer SK, Paul P, Majdic O, Heitger A, Felzmann T (2005). Generation of potent anti-tumor immunity in mice by interleukin-12-secreting dendritic cells. Cancer Immunol Immunother.

[174] Erhart F, Buchroithner J, Reitermaier R, Fischhuber K, Klingenbrunner S, Sloma I (2018). Immunological analysis of phase II glioblastoma dendritic cell vaccine (Audencel) trial: immune system characteristics influence outcome and Audencel up-regulates Th1-related immunovariables. Acta Neuropathol Commun.

[175] Dziurzynski K, Chang SM, Heimberger AB, Kalejta RF, McGregor Dallas SR, Smit M (2012). Consensus on the role of human cytomegalovirus in glioblastoma. Neuro Oncol.

[176] Plon SE, Pirics ML, Nuchtern J, Hicks J, Russell H, Agrawal S (2008). Cytomegalovirus immunity after vaccination with autologous glioblastoma lysate. Cytokine (pg/ml).

[177] Mitchell DA, Batich KA, Gunn MD, Huang M-N, Sanchez-Perez L, Nair SK (2015). Tetanus toxoid and CCL3 improve dendritic cell vaccines in mice and glioblastoma patients. Nature.

[178] Batich KA, Mitchell DA, Healy P, Herndon JE, Sampson JH (2020). Once, twice, three times a finding: reproducibility of dendritic cell vaccine trials targeting cytomegalovirus in glioblastoma. Clin Cancer Res.

[179] Tarhriz V, Eyvazi S, Musavi M, Abasi M, Sharifi K, Ghanbarian H (2019). Transient induction of Cdk9 in the early stage of differentiation is critical for myogenesis. J Cell Biochemis.

[180] Tarhriz V, Wagner KD, Masoumi Z, Molavi O, Hejazi MS, Ghanbarian H (2018). CDK9 regulates apoptosis of myoblast cells by modulation of microRNA-1 expression. J Cell Biochem.

[181] Mahmoudi E, Cairns M (2017). MiR-137: an important player in neural development and neoplastic transformation. Mol Psychiatry.

[182] Guo Y-R, Cao Q-D, Hong Z-S, Tan Y-Y, Chen S-D, Jin H-J (2020). The origin, transmission and clinical therapies on coronavirus disease 2019 (COVID-19) outbreak–an update on the status. Mil Med Res.

[183] Wang L, Liu J, Zhong Z, Gong X, Liu W, Shi L (2016). PTP4A3 is a target for inhibition of cell proliferatin, migration and invasion through Akt/mTOR signaling pathway in glioblastoma under the regulation of miR-137. Brain Res.

[184] Ji Z-G, Jiang H-T, Zhang P-S (2018). FOXK1 promotes cell growth through activating wnt/β-catenin pathway and emerges as a novel target of miR-137 in glioma. Am J Transl Res.

[185] Gao L, Chen B, Li J, Yang F, Cen X, Liao Z (2017). Wnt/β-catenin signaling pathway inhibits the proliferation and apoptosis of U87 glioma cells via different mechanisms. PLoS ONE.

[186] Lässer C, O’Neil SE, Shelke GV, Sihlbom C, Hansson SF, Gho YS (2016). Exosomes in the nose induce immune cell trafficking and harbour an altered protein cargo in chronic airway inflammation. J Transl Med.

[187] Gonzalez-Calero L, Martin-Lorenzo M, Alvarez-Llamas G (2014). Exosomes: a potential key target in cardio-renal syndrome. Front Immunol.

[188] Kishore R, Garikipati VNS, Gumpert A (2016). Tiny shuttles for information transfer: exosomes in cardiac health and disease. J Cardiovasc Transl Res.

[189] Howitt J, Hill AF (2016). Exosomes in the pathology of neurodegenerative diseases. J Biol Chem.

[190] Record M, Poirot M, Silvente-Poirot S (2014). Emerging concepts on the role of exosomes in lipid metabolic diseases. Biochimie.

[191] Kamerkar S, LeBleu VS, Sugimoto H, Yang S, Ruivo CF, Melo SA (2017). Exosomes facilitate therapeutic targeting of oncogenic KRAS in pancreatic cancer. Nature.

[192] Kim SM, Yang Y, Oh SJ, Hong Y, Seo M, Jang M (2017). Cancer-derived exosomes as a delivery platform of CRISPR/Cas9 confer cancer cell tropism-dependent targeting. J Control Release.

[193] Lai CP, Mardini O, Ericsson M, Prabhakar S, Maguire CA, Chen JW (2014). Dynamic biodistribution of extracellular vesicles in vivo using a multimodal imaging reporter. ACS Nano.

[194] McNamara MG, Sahebjam S, Mason WP (2013). Emerging biomarkers in glioblastoma. Cancers.

[195] Alentorn A, Duran-Peña A, Pingle SC, Piccioni DE, Idbaih A, Kesari S (2015). Molecular profiling of gliomas: potential therapeutic implications. Expert Rev Anticancer Ther.

[196] Wang Q, Wang Z, Chu L, Li X, Kan P, Xin X (2015). The effects and molecular mechanisms of MiR-106a in multidrug resistance reversal in human glioma U87/DDP and U251/G cell lines. PLoS ONE.

[197] Ostrom QT, Gittleman H, Liao P, Vecchione-Koval T, Wolinsky Y, Kruchko C (2017). CBTRUS statistical report: primary brain and other central nervous system tumors diagnosed in the United States in 2010–2014. Neuro-Oncology.

[198] Cohen AL, Colman H (2015). Glioma biology and molecular markers .Current understanding and treatment of gliomas. Cancer Treat Res.

[199] Ludwig K, Kornblum HI (2017). Molecular markers in glioma. J Neurooncol.

[200] Yuan Y, Qi C, Maling G, Xiang W, Yanhui L, Ruofei L (2016). TERT mutation in glioma: frequency, prognosis and risk. J Clin Neurosci.

[201] Xu H, Zhao G, Zhang Y, Jiang H, Wang W, Zhao D (2019). Mesenchymal stem cell-derived exosomal microRNA-133b suppresses glioma progression via Wnt/β-catenin signaling pathway by targeting EZH2. Stem Cell Res Ther.

[202] Yu L, Gui S, Liu Y, Qiu X, Zhang G, Xa Zhang (2019). Exosomes derived from microRNA-199a-overexpressing mesenchymal stem cells inhibit glioma progression by down-regulating AGAP2. Aging (Albany NY).

[203] Kim R, Lee S, Lee J, Kim M, Kim WJ, Lee HW (2018). Exosomes derived from microRNA-584 transfected mesenchymal stem cells: novel alternative therapeutic vehicles for cancer therapy. BMB Rep.

[204] Chen X, Yang F, Zhang T, Wang W, Xi W, Li Y (2019). MiR-9 promotes tumorigenesis and angiogenesis and is activated by MYC and OCT4 in human glioma. J Exp Clin Cancer Res.

[205] Guo X, Qiu W, Liu Q, Qian M, Wang S, Zhang Z (2018). Immunosuppressive effects of hypoxia-induced glioma exosomes through myeloid-derived suppressor cells via the miR-10a/Rora and miR-21/Pten Pathways. Oncogene.

[206] Santangelo A, Imbrucè P, Gardenghi B, Belli L, Agushi R, Tamanini A (2018). A microRNA signature from serum exosomes of patients with glioma as complementary diagnostic biomarker. J Neurooncol.

[207] Henriksen M, Johnsen KB, Olesen P, Pilgaard L, Duroux M (2014). MicroRNA expression signatures and their correlation with clinicopathological features in glioblastoma multiforme. NeuroMol Med.

[208] Lang FM, Hossain A, Gumin J, Momin EN, Shimizu Y, Ledbetter D (2018). Mesenchymal stem cells as natural biofactories for exosomes carrying miR-124a in the treatment of gliomas. Neuro Oncol.

[209] van der Vos KE, Abels ER, Zhang X, Lai C, Carrizosa E, Oakley D (2016). Directly visualized glioblastoma-derived extracellular vesicles transfer RNA to microglia/macrophages in the brain. Neuro Oncol.

[210] Soofiyani SR, Hosseini K, Soleimanian A, Abkhooei L, Hoseini AM, Tarhriz V (2021). An overview on the role of mir-451 in lung cancer: diagnosis, therapy, and prognosis. Microrna.

[211] Monteforte A, Lam B, Sherman MB, Henderson K, Sligar AD, Spencer A (2017). Glioblastoma exosomes for therapeutic angiogenesis in peripheral ischemia. Tissue Eng Part A.

[212] Akers JC, Ramakrishnan V, Kim R, Phillips S, Kaimal V, Mao Y (2015). miRNA contents of cerebrospinal fluid extracellular vesicles in glioblastoma patients. J Neurooncol.

[213] Fareh M, Almairac F, Turchi L, Burel-Vandenbos F, Paquis P, Fontaine D (2017). Cell-based therapy using miR-302–367 expressing cells represses glioblastoma growth. Cell Death Dis.

[214] Tűzesi Á, Kling T, Wenger A, Lunavat TR, Jang SC, Rydenhag B (2017). Pediatric brain tumor cells release exosomes with a miRNA repertoire that differs from exosomes secreted by normal cells. Oncotarget.

[215] Figueroa J, Phillips LM, Shahar T, Hossain A, Gumin J, Kim H (2017). Exosomes from glioma-associated mesenchymal stem cells increase the tumorigenicity of glioma stem-like cells via transfer of miR-1587. Can Res.

[216] Deng S-Z, Lai M-F, Li Y-P, Xu C-H, Zhang H-R, Kuang J-G (2020). Human marrow stromal cells secrete microRNA-375-containing exosomes to regulate glioma progression. Cancer Gene Ther.

[217] Qian M, Wang S, Guo X, Wang J, Zhang Z, Qiu W (2020). Hypoxic glioma-derived exosomes deliver microRNA-1246 to induce M2 macrophage polarization by targeting TERF2IP via the STAT3 and NF-κB pathways. Oncogene.

[218] Sharif S, Ghahremani M, Soleimani M (2018). Delivery of exogenous miR-124 to glioblastoma multiform cells by Wharton’s jelly mesenchymal stem cells decreases cell proliferation and migration, and confers chemosensitivity. Stem Cell Reviews Rep.

[219] Shao N, Xue L, Wang R, Luo K, Zhi F, Lan Q (2019). miR-454-3p is an exosomal biomarker and functions as a tumor suppressor in glioma. Mol Cancer Ther.

[220] Katakowski M, Buller B, Zheng X, Lu Y, Rogers T, Osobamiro O (2013). Exosomes from marrow stromal cells expressing miR-146b inhibit glioma growth. Cancer Lett.

[221] Yang J-K, Yang J-P, Tong J, Jing S-Y, Fan B, Wang F (2017). Exosomal miR-221 targets DNM3 to induce tumor progression and temozolomide resistance in glioma. J Neurooncol.

[222] Wang Z-F, Liao F, Wu H, Dai J (2019). Glioma stem cells-derived exosomal miR-26a promotes angiogenesis of microvessel endothelial cells in glioma. J Exp Clin Cancer Res.

[223] Sedighi M, Zahedi Bialvaei A, Hamblin MR, Ohadi E, Asadi A, Halajzadeh M (2019). Therapeutic bacteria to combat cancer; current advances, challenges, and opportunities. Cancer Med.

[224] Pace KR, Dutt R, Galileo DS (2019). Exosomal L1CAM stimulates glioblastoma cell motility, proliferation, and invasiveness. Int J Mol Sci.

[225] Yoon JH, Kim J, Kim KL, Kim DH, Jung SJ, Lee H (2014). Proteomic analysis of hypoxia-induced U373MG glioma secretome reveals novel hypoxia-dependent migration factors. Proteomics.

[226] Zhao C, Wang H, Xiong C, Liu Y (2018). Hypoxic glioblastoma release exosomal VEGF-A induce the permeability of blood-brain barrier. Biochem Biophys Res Commun.

[227] Kore RA, Abraham EC (2014). Inflammatory cytokines, interleukin-1 beta and tumor necrosis factor-alpha, upregulated in glioblastoma multiforme, raise the levels of CRYAB in exosomes secreted by U373 glioma cells. Biochem Biophys Res Commun.

[228] Huang K, Fang C, Yi K, Liu X, Qi H, Tan Y (2018). The role of PTRF/Cavin1 as a biomarker in both glioma and serum exosomes. Theranostics.

[229] Madhankumar A, Mrowczynski OD, Patel SR, Weston CL, Zacharia BE, Glantz MJ (2017). Interleukin-13 conjugated quantum dots for identification of glioma initiating cells and their extracellular vesicles. Acta Biomater.

[230] Svensson KJ, Christianson HC, Wittrup A, Bourseau-Guilmain E, Lindqvist E, Svensson LM (2013). Exosome uptake depends on ERK1/2-heat shock protein 27 signaling and lipid Raft-mediated endocytosis negatively regulated by caveolin-1. J Biol Chem.

[231] Setti M, Osti D, Richichi C, Ortensi B, Del Bene M, Fornasari L (2015). Extracellular vesicle-mediated transfer of CLIC1 protein is a novel mechanism for the regulation of glioblastoma growth. Oncotarget.

[232] Pinet S, Bessette B, Vedrenne N, Lacroix A, Richard L, Jauberteau M-O (2016). TrkB-containing exosomes promote the transfer of glioblastoma aggressiveness to YKL-40-inactivated glioblastoma cells. Oncotarget.

[233] Bai H, Pan Y, Qi L, Liu L, Zhao X, Dong H (2018). Development a hydrazide-functionalized thermosensitive polymer based homogeneous system for highly efficient N-glycoprotein/glycopeptide enrichment from human plasma exosome. Talanta.

[234] Kore RA, Edmondson JL, Jenkins SV, Jamshidi-Parsian A, Dings RP, Reyna NS (2018). Hypoxia-derived exosomes induce putative altered pathways in biosynthesis and ion regulatory channels in glioblastoma cells. Biochemis Biophys Rep.

